# Evaluation of fecal microbiota of late gestation sows in relation to pelvic organ prolapse risk

**DOI:** 10.3389/fmicb.2024.1384583

**Published:** 2024-07-24

**Authors:** Zoë E. Kiefer, Lucas R. Koester, Jamie M. Studer, Stephan Schmitz-Esser, Jason W. Ross

**Affiliations:** ^1^Department of Animal Science, Iowa State University, Ames, IA, United States; ^2^Department of Veterinary Microbiology and Preventive Medicine, Iowa State University, Ames, IA, United States; ^3^Interdepartmental Microbiology Graduate Program, Iowa State University, Ames, IA, United States

**Keywords:** pelvic organ prolapse, fecal microbiota, vaginal microbiota, reproduction, sow

## Abstract

**Introduction:**

Sow mortality in the U.S. swine industry has increased in recent years, for which pelvic organ prolapse (POP) is a major contributor, accounting for 21% of all sow mortality. Dysbiosis of microbial communities has been associated with disease and reproductive dysfunction in several species, and previous studies have shown changes in vaginal microbiota in sows with increased risk for POP during late gestation. However, there is insufficient knowledge surrounding the potential relationship between fecal microbiota and POP in sows. Therefore, the study objective was to identify differences in sow fecal microbiota and determine if fecal and vaginal microbial communities are correlated in relation to POP risk.

**Methods:**

Sows were evaluated for POP risk using an established perineal scoring system, with a perineal score (PS) of 1 (PS1) presuming little to no risk of POP to a PS of 3 (PS3) presuming high risk of POP. In the current study, 2,864 sows were scored during gestation week 15, and 1.0%, 2.7%, and 23.4% of PS1, PS2, and PS3 sows, respectively, subsequently experienced POP. Fecal swabs (*n* = 215) were collected between gestation days 108-115, DNA was extracted, and 16S rRNA gene amplicon sequencing libraries were analyzed using mothur, phyloseq and SAS in reference to PS and POP outcome. Additionally, co-occurrence networks were constructed using CoNet to compare fecal and vaginal microbiota from the same cohort of sows and identify correlations between different taxa.

**Results:**

Differences in fecal community composition (PERMANOVA; *P* < 0.05), structure (alpha diversity measurements; *P* < 0.05), and 13 individual operational taxonomic units (OTUs) were revealed between PS1 and PS3 assigned sows. No differences in fecal microbiota were detected as a result of POP outcome. However, the abundances of several taxa were correlated across sample collection sites, suggesting the fecal and vaginal microbial communities may be related to one another.

**Discussion:**

Collectively, fewer differences in the fecal microbiota exist in sows with differing risk for POP compared to the vaginal microbiota, suggesting the vaginal microbiome may be more relevant in relation to POP outcome, although correlations between fecal and vaginal communities may provide insight for strategies to combat POP.

## Introduction

Over the last 50 years, substantial progress has been made in efficiency of swine production, including improvements in reproduction. Despite these improvements, sow mortality has increased throughout the last decade with up to 21% (Ross, 2019) due to pelvic organ prolapse (POP). Typically considered an anatomical disorder, POP is characterized by one or more of the pelvic organs pressing up against or out of the vagina (Jelovsek et al., 2007), and sows most commonly experience POP during late gestation or shortly after farrowing (Supakorn et al., 2014). Although recent work has begun to elucidate potential underlying mechanisms contributing to POP, a definitive understanding of the biological causes of POP is lacking. As POP is not only an economic issue but also an animal welfare concern, further investigation is needed to develop mitigation strategies that effectively reduce prevalence of POP in the U.S. sow herd.

Microbial communities of a host can influence host health, and dysbiosis in these microbial communities can indicate dysfunction at a particular site within a host (Petersen and Round, 2014). In recent years, studies have begun to explore associations between gut, fecal, and vaginal microbiota in relation to swine growth, health, and reproduction (Kamada et al., 2012; Hermann-Bank et al., 2015; Yuliaxis et al., 2016; Wang et al., 2017; Yang et al., 2018; Wang et al., 2019; He et al., 2020; Sanglard et al., 2020a,b; Zhong et al., 2021; Kiefer et al., 2021a,b). However, due to a lack of understanding and research in this area, it is difficult to differentiate a healthy sow vaginal microbiome from one experiencing dysbiosis. Previous studies have demonstrated that sows with differing risk for POP have notable differences in the vaginal microbiota, and several relevant microbial taxa have been identified as potential biomarkers for disease (Kiefer et al., 2021a,b). To our knowledge, the relationship between POP and fecal microbiota has not yet been analyzed in late gestation sows, therefore further investigation is warranted.

Interestingly, studies have recently revealed that gut microbial communities play a role in estrogen metabolism, and changes in these communities can affect steroid hormone levels (Adlercreutz et al., 1984; Cotton et al., 2023). Additionally, studies conducted in humans have demonstrated that the composition of the gut microbiota has an effect on circulating estrogens and estrogen metabolite levels (Flores et al., 2012; Baker et al., 2017). Similarly, studies conducted in female mice have reported that sex hormones may affect the gut microbial communities (Acharya et al., 2019). Previously, we have identified differences in steroid hormone levels in sows with differing risk for POP (Kiefer et al., 2021c), but the relationship between these hormone levels and the fecal microbiota has not been studied. Similarly, the relationship and potential interaction between these communities in relation to POP risk has yet to be explored.

Furthermore, studies have begun to evaluate correlations between microbiomes at different body sites (Xu et al., 2021) and their relationship to reproductive health (Deng et al., 2019; Zhang et al., 2021; Gu et al., 2022). Given the close physical proximity of the external genitalia (anus and vulva) and shared urogenital tract in female pigs, the inherent anatomical structure of the sow may enable a connection between the fecal and vaginal microbial communities. Therefore, the potential influence these communities have on one another is understudied despite the evidence linking both vaginal and fecal microbiota to reproductive health.

Previously, alterations in vaginal microbiota and circulating hormone levels between sows differing in POP risk have been observed (Kiefer et al., 2021c). These observations, in addition to the new developments surrounding the relationship between microbial communities and reproductive hormone levels, warrants additional research to investigate if alterations in fecal microbiota contribute to risk for experiencing POP. One aim of the study was to identify whether or not the fecal microbiota could be utilized as an indicator of POP risk. Thus, the objective of this study was to identify differences in fecal microbiota of late gestation sows, determine if the fecal and vaginal microbial communities are related, and determine if these differences or correlations are associated with POP risk. To accomplish this, the current study tested the hypothesis that fecal microbiota differs between sows at variable risk for POP. Additionally, we aimed to identify correlations in microbial taxa between the vaginal and fecal microbiota to provide a deeper understanding of the similarities between these two communities in swine.

## Materials and methods

### Animals

A total of 2,864 sows during late gestation (days 108–115) were included in this trial. All experiments involving animals were approved by the Iowa State University (ISU) Institutional Animal Care and Use Committee. This work was conducted on two commercial sow farms, designated farm A and farm B, from the same production system. These farms were in close proximity to one another and shared the same genetics, feed, and housing type. Within each farm, sows were housed in individual gestation stalls in the same room, with sows grouped by date of first service. Additionally, the farms had a similar health status being porcine reproductive and respiratory syndrome naïve, *Mycoplasma hyopneumoniae* stable, porcine epidemic disease naïve, and influenza A virus stable.

### Perineal scoring system

Three perineal score (PS) classes, varying from presumed low to high risk, were used to classify the late gestation sows for POP risk, and has been described previously (Kiefer et al., 2021a). Sows were only assigned a PS while lying down. In brief, to assign the PS, the perineal region was visually evaluated for swelling, redness, and protrusion. A sow lacking swelling, redness, and protrusion was assigned a PS1 and considered low risk for POP. Sows with moderate swelling, redness, and protrusion of the perineal area were assigned a PS2, assuming moderate risk for POP. Sows assigned PS3 demonstrated all the characteristics of severe swelling, redness, and protrusion of the perineal area and were considered high risk for POP. Scores were assigned over 7 consecutive weeks during Spring of 2019 during gestation days 108–115, with sows on each farm being scored once each week on subsequent days as previously documented (Kiefer et al., 2021b).

### Statistical analysis of perineal score

The main effects of PS and Farm, both of which are considered categorical, and the interaction between PS and Farm, Parity, and the interaction between PS and Parity were assessed in SAS 9.4 (Cary, NC) utilizing a PROC MIXED analysis. Data are considered significant if *p* ≤ 0.05 and indicate a tendency for biological meaning if 0.05 < *p* < 0.10.

### Sample collection

This study is an expansion of prior work investigating differences in blood parameters (Kiefer et al., 2021c) and vaginal microbiota (Kiefer et al., 2021b) between PS1 and PS3 sows. At both farms, fecal swabs were collected in tandem with the vaginal swabs from the previous study (Kiefer et al., 2021b) during gestation week 15 (days 108–115). All sows classified as PS3 (*n* = 117) were swabbed along with parity-matched PS1 (*n* = 96) assigned sows. Of the 117 PS3 assigned sows, 28 of them experienced POP with 9 being from Farm A and 19 being from Farm B. None of the PS1 sows evaluated experienced POP. Because some sows are moved from gestation to farrowing rooms during the late stages of gestation (approximately day 112 to 113 of gestation), some collections occurred in the farrowing room. However, PS1 sow sample collection was matched for parity and day of gestation, making fecal swab collections balanced across room locations for each PS. Fecal swabs were collected by aseptically inserting a 7-inch histology brush (2199, Puritan Medical Products) into the rectum and brushing the orifice for approximately 15 s. Swabs were removed, immediately placed in sterile 1X phosphate buffered saline (PBS), and maintained on wet ice for 12–24 h before being transported to the lab for processing. Due to biosecurity measures on the farms, limited supplies were able to brought onto site during sample collection, for which the authors acknowledge the potential of bias by storage on ice as opposed to immediately freezing, however, all samples were consistently collected and stored until processing (Tedjo et al., 2015; Bassis et al., 2017). Sample processing involved vortexing swabs for 5 min to detach cells, followed by centrifugation at 5,000 × g for 15 min at room temperature to form a pellet. Supernatant was discarded and pellets were stored at −80°C until used for DNA extraction.

### DNA extraction

Fecal microbiota pellets were thawed and DNA was extracted using the Qiagen DNeasy Powerlyzer Powersoil kit (Qiagen, Germantown, MD) per manufacturer’s protocol. Mechanical cell lysis was performed using a Beadmill 24 (Fisher Scientific, Hampton NH). Concentration of isolated DNA was determined using a Qubit^®^ 3.0 Fluorometer (Fisher Scientific, Hampton NH).

### 16S rRNA gene sequencing

16S rRNA gene sequences were amplified from fecal samples of individual sows. DNA was diluted in sterile water to a concentration between 25–75 ng/μL and sent to the ISU DNA facility for sequencing using the Illumina MiSeq platform (Illumina, San Diego, CA, United States). Briefly, the genomic DNA from each sample was amplified using Platinum^™^ Taq DNA Polymerase (Thermo Fisher Scientific, Waltham, MA, United States) with one replicate per sample using universal 16S rRNA gene bacterial primers [515F (5′-GTGYCAGCMGCCGCGGTAA-3′; 26) and 806R (5′-GGACT ACNVGGGTWTCTAAT-3′; 27)] amplifying the variable region V4, as previously described (Kozich et al., 2013). All samples underwent PCR with an initial denaturation step at 94°C for 3 min, followed by 45 s of denaturing at 94°C, 20 s of annealing at 50°C, and 90 s of extension at 72°C. This was repeated for 35 total PCR cycles and finished with a 10-min extension at 72°C. All PCR products were then purified with the QIAquick 96 PCR Purification Kit (Qiagen, Hilden, Germany) according to the manufacturer’s instructions. PCR bar-coded amplicons were mixed at equal molar ratios and used for Illumina MiSeq paired-end sequencing with 250 bp read length and cluster generation with 10% PhiX control DNA.

### Quality control and clustering of sequences into OTUs

Sequence analysis was performed with mothur V1.43.0 following the mothur MiSeq Standard Operating Procedure (Kozich et al., 2013). Barcode sequences, primers and low-quality sequences were trimmed using a minimum average quality score of 35, with a sliding window size of 50 bp. Chimeric sequences were removed using the “Chimera.vsearch” command. For alignment and taxonomic classification of OTUs, the Silva SSU NR reference database (v138) provided by the mothur website was used. Sequences were clustered into OTUs with a cutoff of 99% 16S rRNA gene similarity (= 0.01 distance) and were ordered from most to least abundant. Representative sequences for the 50 most abundant OTUs were additionally classified using NCBI BLAST (Altschul et al., 1990) to improve classification accuracy.

### Microbial sequence analysis

Samples were assigned a Bray–Curtis dissimilarity value and means were compared using the permutational analysis of variance (PERMANOVA) package built in to Phyloseq. Bray–Curtis dissimilarity was selected based on the ability to compare closely-related samples (Bray and Curtis, 1957).

Measurements of Chao species richness, Shannon Diversity, and Simpson evenness were conducted to compare community structures (alpha diversity) between experimental groups. The means of the experimental group alpha diversity measures were analyzed using the PROC MIXED procedure in SAS. To compare overall microbial community composition (beta diversity), Bray–Curtis dissimilarity measurements between experimental groups were analyzed using the Adonis command (PERMANOVA) and BetaDisp command (BetaDispersr) provided within the VEGAN v2.5-5 package (Oksanen et al., 2020). Overall variation in bacterial communities was visualized using principal coordinate analysis (PCoA). Canonical analysis of principal coordinates (CAP) (Anderson and Willis, 2003) was used to visualize the variation capture by PS, Farm, and the interaction between the two. This information was generated with the Phyloseq v1.34.0 (McMurdie and Holmes, 2013) and Vegan. All plotting was completed using ggplot2, v2_3.1.1 graphing package in R 4.1.0.

The absolute abundances of the 100 most abundant OTUs among samples were analyzed using a negative binomial distribution in GLIMMIX procedure of SAS (Version 9.4, SAS Inst., Cary, NC), and were offset by the total library count for a given sample. Corresponding *p*-values were corrected for false discovery rates (FDR) using the FDR correction of the MULTITEST procedure within SAS. For all comparisons, *p* (or *Q*) values were considered significant if <0.05, and trending towards significance if 0.05 < *p* (or *Q*) < 0.10. For the top 100 OTUs with a *Q* value of ≤0.05, the Log2-fold changes (log2FC) between groups were calculated using R and plotted using ggplot2. Three analyses were conducted; (1) PS1 (*n* = 96) sows compared to PS3 (*n* = 117) sows, (2) comparison between all sows that did (Yes; *n* = 28) or did not (No; *n* = 185) subsequently experience POP, and (3) comparison between PS3 sows that did (Yes; *n* = 28) or did not (No; *n* = 89) subsequently experience POP.

Additionally, the vaginal microbial sequencing data from Kiefer et al. (2021b) and the current study were combined and processed together in mothur and phyloseq, using the pipeline described above, to determine shared microbial populations. These studies were conducted in parallel on the same sow farms utilizing the same cohort of sows for sample collection. Shared OTUs between studies were calculated and visualized using the VennDiagram package in R. Overall community structure was visualized using PCoA plots generated in phyloseq (see above) and overall community differences were tested using PERMANOVA. Finally, co-occurrence network analysis was conducted using the 100 most abundant OTUs to identify correlations between fecal and vaginal microbiota using CoNet v.1.1.1 in Cytoscape (Faust and Raes, 2016). In these networks, the nodes represent microbial species and the edges represent significant (*p* < 0.05) relationships in abundance patterns. Networks for within and between body sites were created, evaluating both negative (red) and positive (green) correlations, and were only considered significant if correlations existed between at least two tests.

## Results

### Changes in perineal score throughout late gestation

All data involving PS and POP incidence has been previously described in Kiefer et al. (2021b,c). In total, 2,864 sows were scored across both farms during gestation week 15. A difference (*p* < 0.01) in POP incidence was observed between PS1, PS2, and PS3 sows, with 1.0% of PS1, 2.7% of PS2, and 23.4% of PS3 scored sows, subsequently experiencing POP. Additionally, Farm A had a 1.6% POP rate while Farm B had a 3.7% POP rate during the duration of this study. Of all sows scored at both farms, PS was affected (*p* < 0.01) by parity with sows assigned PS1 having an average parity of 1.9 ± 0.1 compared to 3.3 ± 0.2 for PS3 scored sows, which has been previously described in Kiefer et al. (2021b). To account for differences due to parity, samples were collected from PS3 sows along with parity matched PS1 sows to be used for microbiota sequencing. In this subset of sows, the average parity of PS1 sows was 3.0 ± 0.2 while the average parity of PS3 sows was 3.3 ± 0.2.

### Characterization of the fecal microbiota of late gestation sows

A total of 137,933 OTUs were obtained from 213 fecal samples of which 8,224 OTUs remained after removal of OTUs represented by less than 10 sequence reads. The average sequencing depth per sample was 36,788 sequences with a standard deviation of 10,654 sequences. Bacterial reads made up 99.05% of the total reads while 0.95% were archaeal. Across the 8,224 OTUs used in this dataset, 26 and 457 unique phyla and genera, respectively, were represented. The 50 most abundant fecal OTUs are reported in Supplementary Table S1.

### Differences in fecal microbiota exist between farms and sows at varying risk of POP during week 15 of gestation

**Figure 1:**
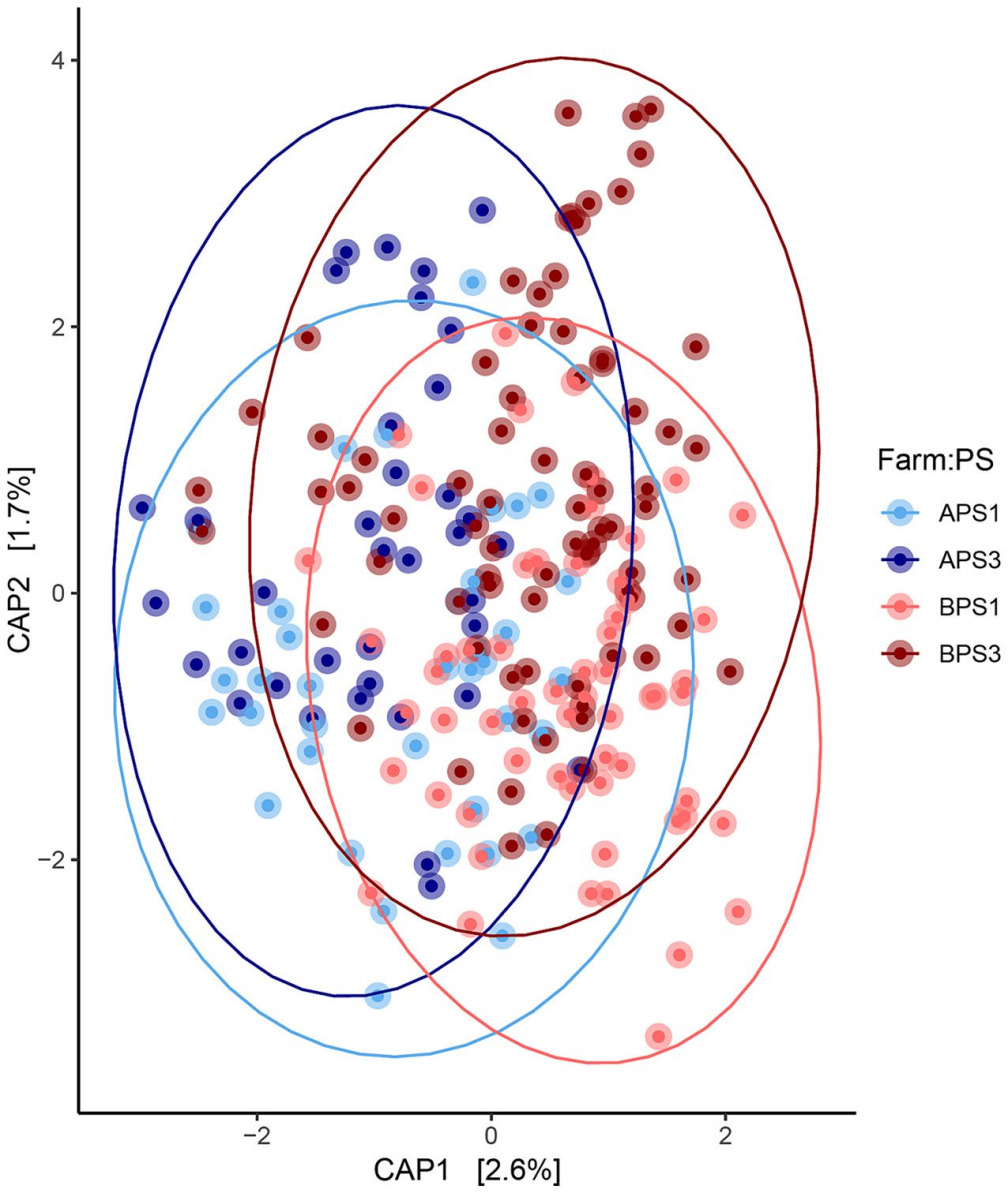
Fecal microbial community comparisons. Canonical analysis of principal coordinates (CAP) demonstrating the maximum variation of beta-diversity between fecal microbiota communities. The CAP from sows with assumed low (PS1, *n* = 96) or high (PS3, *n* = 117) risk for pelvic organ prolapse (POP) during gestation week 15 (days 108–115) from two separate farms (A and B). Farm A, PS1 (APS1; light blue), Farm A, PS3 (APS3; dark blue); Farm B, PS1 (BPS1; light red) and Farm B, PS3 (BPS3; dark red). Statistical differences (*p* < 0.01) were detected in overall microbial communities between perineal score (PS) and Farm using PERMANOVA.

**Figure 2:**
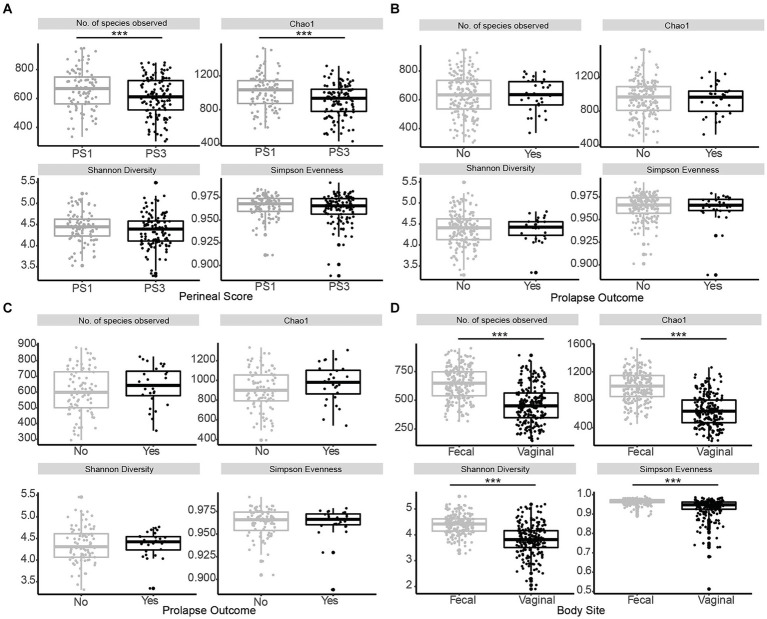
Alpha diversity of fecal microbiota in late gestation sows. Comparison of alpha diversity measurements species evenness (Simpson), richness (number of species observed, Chao1), and diversity (Shannon) across the variables within this study. **(A)** Alpha diversity measurements for fecal microbiota of sows with low (PS1; grey, *n* = 96) or high (PS3; black, *n* = 117) risk for pelvic organ prolapse (POP) at gestation week 15 (days 108–115) revealed significant differences (*p* ≤ 0.01) in community structure between samples regarding Chao species richness and the number of observed species. **(B)** Alpha diversity measurements from sows that subsequently did (Yes; black, *n* = 28) or did not (No; grey, *n* = 185) experience POP revealed no differences (*p* ≥ 0.66) in community structure. **(C)** Alpha diversity measurements for PS3 sows that subsequently did (Yes; black, *n* = 28) or did not (No; grey, *n* = 89) experience POP revealed no differences (*p* ≥ 0.14) in community structure. **(D)** Alpha diversity measurements for fecal microbiota compared to vaginal microbiota of all sows at gestation week 15. Species evenness (Simpson), richness (number of species observed, Chao1), and diversity (Shannon) were different (*p* < 0.01) between body sites. Significance is noted by ^***^p ≤ 0.05.

When evaluating fecal microbiota on a whole-community level at gestation week 15, differences (*p* ≤ 0.01) were detected between PS and Farm, and the interaction of PS and Farm had a tendency (*p* = 0.07) to be different. When evaluating community separation using CAP, a difference (*p* < 0.01) was observed between PS and Farm (Figure 1). Alpha diversity estimators revealed differences (*p* ≤ 0.01) in community structure between samples regarding Chao species richness and total number of observed species (Figure 2A). When evaluating the 100 most abundant OTUs, differences (*p* < 0.05, *Q* < 0.05) in abundance of 13 OTUs were observed (Table 1). Increases in *Turicibacter* (OTU 1), *Clostridium_sensu_stricto_1* (OTU 2, 3, and 5), *Anaerococcus* (OTU 7), *Treponema* (OTU 20), *Actinobacillus* (OTU 38), *Firmicutes_unclassified* (OTU 40), *Streptococcus* (OTU 51), *Helicobacter* (OTU 59), and *Oscillospiraceae* (OTU 76) were observed in PS3 sows compared to PS1 sows. In the fecal microbiota of PS1 sows, both *Ezakiella* (OTU 46) and *Anaerococcus* (OTU 94) were increased (*p* < 0.01) compared to PS3 sows.

**Table 1:** Differences in OTUs between fecal microbiota of PS1 and PS3 sows during gestation week 15.

OTU^a^	Taxonomy (Silva v138)^b^	NCBI BLAST				
		Classification	Similarity (%)	PS^c^	Log2FC^d^	*p-*value
OTU 1	*Turicibacter*	*Turicibacter* sp. H121	99.6	PS3	0.23	0.002
OTU 2	*Clostridium_sensu_stricto_1*	*Clostridium* sp.	98.0	PS3	0.24	0.002
OTU 3	*Clostridium_sensu_stricto_1*	*Clostridium moniliforme*	99.6	PS3	0.25	0.002
OTU 5	*Clostridium_sensu_stricto_1*	*Clostridium celatum*	100	PS3	0.24	0.002
OTU 7	*Anaerococcus*	*Anaerococcus tetradius*	100	PS3	0.39	0.00003
OTU 20	*Treponema*	*Treponema bryantii*	99.6	PS3	0.40	0.0002
OTU 38	*Actinobacillus*	*Terrahaemophilus aromaticivorans*	99.6	PS3	0.69	0.0004
OTU 40	*Firmicutes_unclassified*	*Bacterium*	92.5	PS3	0.32	0.002
OTU 46	*Ezakiella*	*Ezakiella coagulans*	97.6	PS1	0.77	0.0008
OTU 51	*Streptococcus*	*Streptococcus parasuis*	99.6	PS3	0.66	0.0003
OTU 59	*Helicobacter*	*Helicobacter equorum*	99.6	PS3	0.51	0.002
OTU 76	*Oscillospiraceae* NK4A214_group	*Oscillospiraceae bacterium*	95.3	PS3	0.30	0.005
OTU 94	*Anaerococcus*	*Anaerococcus provencensis*	93.7	PS1	1.07	0.004

a Individual microbes were assigned in order of abundance and classified into operational taxonomic units (OTUs).

b Taxonomy was assigned using Silva SSU NR reference database (v138).

c Sows were assigned a perineal score (PS) based on their relative risk of experiencing a pelvic organ prolapse (POP). Sows assigned PS1 were presumed low risk for POP and sows assigned PS3 were presumed high risk for POP. Specific OTUs were more abundant in sows with the PS indicated.

d Log2 fold change.

### Differences in fecal microbiota were not observed between sows that subsequently did or did not experience POP

Whole-community level analysis of the fecal microbiota at gestation week 15 revealed no differences (*p* ≥ 0.42) between POP outcome or the interaction between POP outcome and Farm. Alpha diversity estimators revealed no differences (*p* ≥ 0.66) in community structure between fecal samples regarding Chao species richness and Observed species (Figure 2B). When evaluating the 100 most abundant OTUs no differences in abundance (*p* > 0.05, *Q* > 0.05) were observed. Additionally, whole community level analysis of the fecal microbiota from PS3 sows that subsequently did or did not experience POP revealed no differences (*p* ≥ 0.81) between POP outcome or the interaction between POP outcome and Farm. Alpha diversity estimators revealed no differences (*p* ≥ 0.14) in community structure between samples regarding Chao species richness and total number of observed species (Figure 2C). When comparing the 100 most abundant OTUs no differences in abundance (*p* > 0.05, *Q* > 0.05) were observed. The five most abundant OTUs when evaluating POP included *Turicibacter* and three different *Clostridium*, and *Romboutsia*.

### Comparison of the vaginal and fecal microbial communities

**Figure 3:**
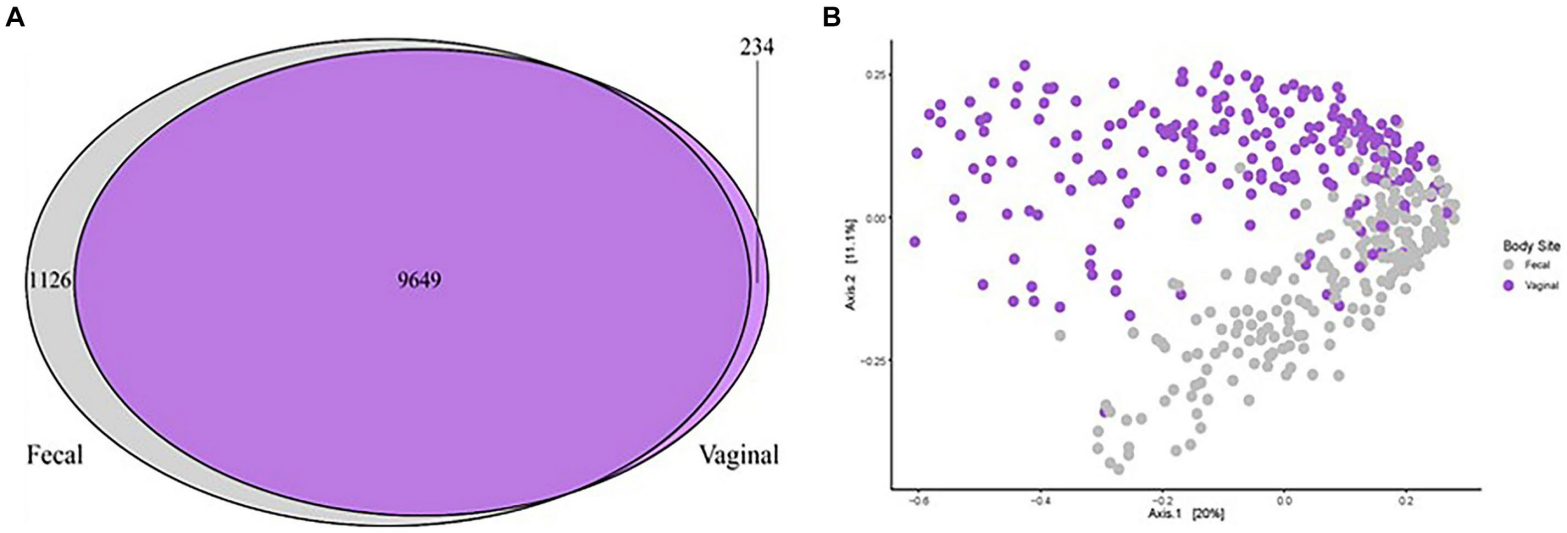
Microbial community comparison analyzing the fecal and vaginal microbiota of sows in relation to POP. **(A)** Venn diagram illustrating the similarities and differences between all vaginal microbiota (Kiefer et al., 2021b, purple) and all fecal microbiota (current study, grey). A total of 9,647 OTUs were shared across studies and an additional 234 were unique to the vaginal communities while 1,128 were unique to the fecal communities. **(B)** Principal coordinates analysis (PCoA) demonstrating differences in beta diversity of all vaginal microbial communities (purple dots) of sows compared to the fecal microbial communities (grey dots). All points represent Bray Curtis dissimilarity measures for each sample. Significant differences (*p* < 0.01) were detected based on sample type (vaginal or fecal).

One aim of the study was to compare the vaginal and fecal microbiota of sows in this trial. When evaluating the microbial communities of both vaginal [data from our previous study (Kiefer et al., 2021b)] and fecal communities on a whole community level at gestation week 15, differences (*p* < 0.01) in community composition were detected (Figure 3B). Vaginal and fecal microbial communities shared the majority of OTUs. However, some of these OTUs had different relative abundances depending on body site. Significant differences (*p* < 0.01) in alpha diversity measurements of species evenness, species richness and species diversity were detected between body sites with the vaginal microbiota showing lower alpha diversity than the fecal microbiota (Figure 2D). A total of 9,649 (87.6%) OTUs were shared across both vaginal and fecal samples, while vaginal samples had 234 (2.1%) unique OTUs and fecal samples had 1,126 (10.2%) unique OTUs (Figure 3A).

### Correlations exist between fecal and vaginal microbiota of late gestation sows may exist

Network analyses were performed to evaluate correlations between the fecal and vaginal microbiota. When comparing the fecal and vaginal communities, 145 nodes, representing 145 individual OTUs, were identified as significantly correlated (*p* < 0.05) and were connected by 485 edges representing correlations between nodes (Supplementary Figure S1 and Supplementary Table S2). Twelve of these edges were negative correlations and the remaining 473 were positive.

Correlations were then calculated between taxa within each environment, resulting in detection of 81 nodes connected by 309 edges, and 64 nodes connected by 160 edges within the fecal and vaginal environments, respectively. Numerous OTUs classified within specific genera of interest were identified as significantly (*p* < 0.05) correlated with several other genera. Some microbes of interest, in relation to PS, within both the fecal and vaginal communities include *Clostridium*, *Treponema*, and *Streptococcus* (associated with increased POP risk), and *Veillonella* (associated with decreased POP risk).

**Figure 4:**
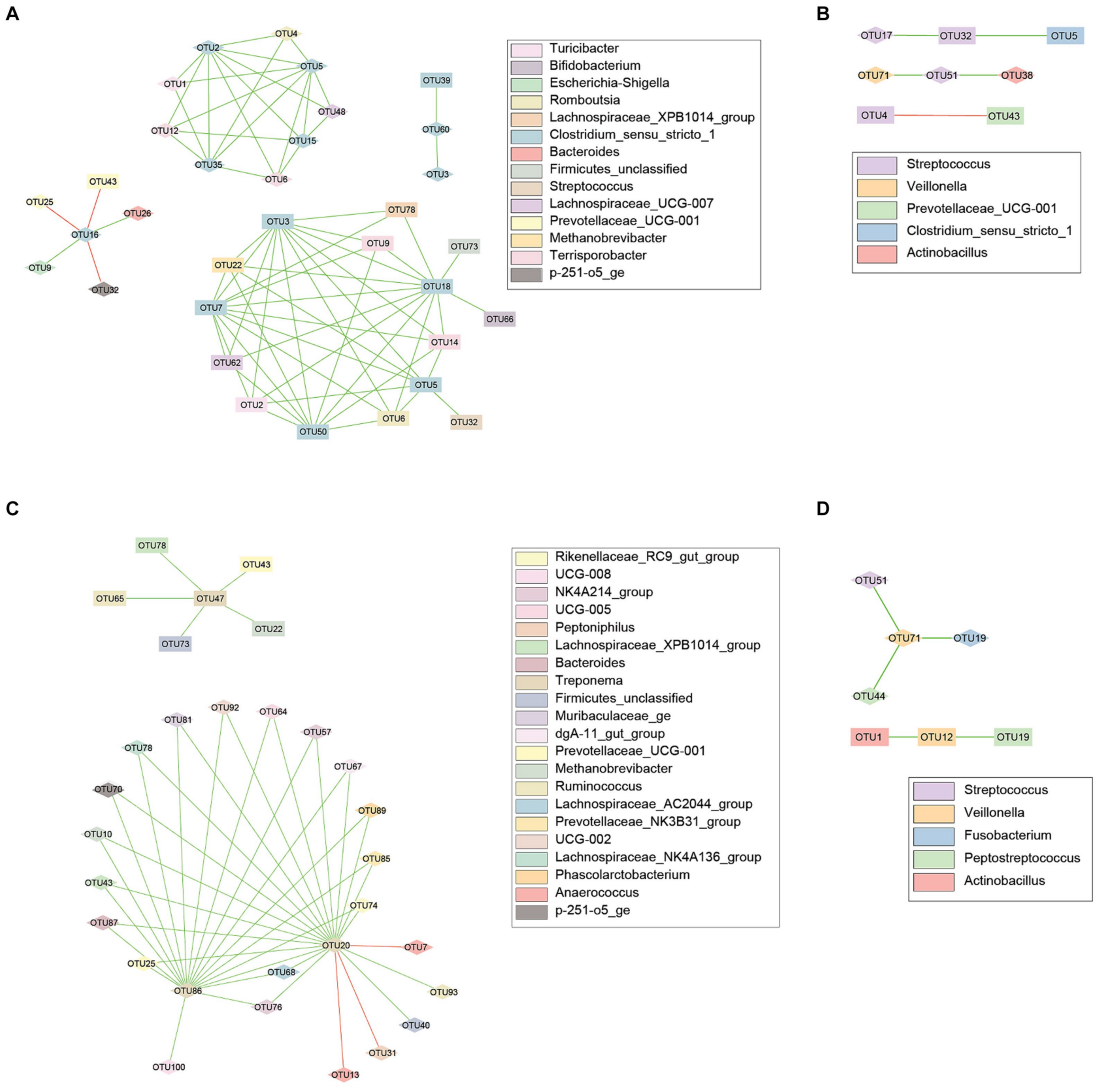
Co-occurrence networks for the fecal and vaginal microbiota of late gestation sows. Co-occurrence networks created using the CoNet (v 1.1.1) application with Cytoscape showing the significant (*p* < 0.05) correlations between the top 100 most abundant OTUs from the fecal and vaginal microbiota of late gestation sows. Nodes represent each individual OTU, with the color representing taxonomy and the shape representing body site (Square = Vaginal, Diamond = Fecal). Edges in green are positive correlations while those in red are negative. **(A)** Evaluated correlations with *Clostridium* species, has 33 nodes correlated, connected by 71 edges. **(B)** 8 nodes were connected by 5 edges between *Streptococcus* species. **(C)** *Treponema* species were connected by 44 edges and included 30 nodes. **(D)** *Veillonella* taxa had only 7 nodes connected by 5 edges.

Lastly, correlations were evaluated between and within body sites. Within the genus *Clostridium*, 33 nodes were observed to be correlated, connected by 71 edges, 3 of which were negatively correlated (Figure 4A). *Streptococcus* had 8 nodes connected by 5 edges, 1 of which was negative (Figure 4B). Thirty nodes were observed within the genus *Treponema*, connected by 44 edges with 2 being negative (Figure 4C). *Veillonella* had 7 nodes connected by 5 edges, all of which were positive (Figure 4D).

## Discussion

Previous studies have demonstrated that vaginal microbiota differs between late gestation sows at high risk for POP compared to vaginal microbiota of low-risk sows (Kiefer et al., 2021a,b). Results from the current study suggest fewer differences exist in the fecal microbiota between sows at variable risk for POP. While the fecal microbiota appears to be less indicative of POP risk, correlations to the vaginal microbiota and differences in community structure were observed between these communities in the same cohort of animals. Collectively, these findings suggest vaginal microbiota may be more relevant to understanding POP than fecal microbiota.

Current literature regarding fecal microbiota in gestating sows is scarce, making it challenging to distinguish a healthy microbial population compared to a population in dysbiosis. Therefore, in addition to exploring fecal microbiota in relation to POP, this study provides novel characterization of the fecal microbiome in sows, and contributes to the growing, but limited, body of knowledge in this field. Overall, 8,224 unique OTUs were identified within the fecal communities of late gestation sows. This is substantially greater than the amount of OTUs previously identified in vaginal (Kiefer et al., 2021a,b) and gut (Wang et al., 2018) microbiota, indicating higher microbial diversity in fecal communities. Additional studies evaluating OTUs in fecal communities of swine have reported a wide range of observations (Kim et al., 2015; Wang et al., 2019), potentially due to low sample sizes, differences in breed and age of animals, or differences in analysis approaches.

Therefore, an objective of the current study was to determine if differences exist in the fecal microbiota of sows at low risk for POP compared to those at high risk. Identifying differences could provide a better understanding of the core fecal microbiota and the specific relationship to POP risk. From this study, 13 OTUs were identified as significantly different when comparing fecal microbiota of PS3 and PS1 sows, despite that the changes in abundance were relatively small. Of these 13 OTUs, 11 were more abundant in high-risk sows and 2 were more abundant in low-risk sows. Specifically, differences were observed in abundances of *Clostridium*, *Anaerococcus*, *Treponema*, *Actinobacillus*, *Ezakiella*, and *Streptococcus*, all of which have been previously identified in sow vaginal microbiota in relation to POP risk (Kiefer et al., 2021a,b). Redundancy of differentially abundant OTUs between the fecal and vaginal communities in relation to POP risk may suggest these specific OTUs are involved in the phenotypic manifestation of a sow with elevated risk of POP, although further research is needed to determine causation.

Recently, it has become widely accepted that the gut microbiome is associated with host health, and changes in this community have been linked to diet, diseases, and host genetics (Duncan et al., 2008). Fecal microbial communities also change depending on the physiological stage of the animal, with gestating or lactating sows having a different profile than other stages (Kim et al., 2015). Further, the gut microbiota of the sow changes throughout pregnancy based on metabolic state (Cheng et al., 2018; Liu et al., 2019). Studying these changes may help characterize a healthy microbial community versus one in dysbiosis, as well as potentially link the causes of dysbiosis to reproductive disorders.

Metabolites produced by the gut microbiota are involved in regulation of host metabolism and health. Specifically, microbially-produced short-chain fatty acids (SCFA), including acetate, propionate, and butyrate, are involved in secretion and regulation of hormones (Ma et al., 2020). Microbes have the ability to degrade hormones, resulting in alterations to circulating estrogen levels and disruption of gut microbiome (Zhong et al., 2021; Cotton et al., 2023). Previously our group has reported differences in concentrations of circulating steroid hormones between sows at high or low risk for POP (Kiefer et al., 2021c). Estrogen has connections to both reproductive and gut health, as well as interactions with microbial communities (Baker et al., 2017). Microbially produced SCFAs have also been reported to regulate prolactin production (Wang et al., 2013), further establishing the involvement of gut microbiota in hormone metabolism. Additionally, production of butyrate by gut microbiota is essential for maintenance of intestinal barrier integrity, and decreases in butyrate production contribute to increased gut permeability and endotoxemia, ultimately triggering low grade inflammation and metabolic disorders (Koh et al., 2016; Cheng et al., 2018; Ma et al., 2020). Endotoxemia occurs when lipopolysaccharide (LPS) endotoxins leak into systemic circulation due to compromised intestinal barrier integrity. Increased LPS-binding protein (LBP) has been observed in circulation of sows at high risk for POP (Kiefer et al., 2021c). Previously we have identified increased abundance of 2-aminobutanoic acid, a butyric acid derivative associated with inflammation, in circulation of PS3 sows compared to PS1 (Kiefer et al., 2021a). In the current study, *Firmicutes*, a phylum containing many known butyrate producers, was increased within fecal microbiota of sows at higher risk for POP. While butyrate is thought to have a potentially protective effect on gut integrity, other confounding influences, such as presence of LPS or other factors, may collectively alter intestinal integrity affecting the ability of microbially produced metabolites to enter the bloodstream. These data support the importance of the gut microbiota and microbially produced metabolites, such as SCFAs, in hormone regulation and host metabolism. Further research is needed to better understand the hormone gut axis and the relationship to microbiota and POP incidence in sows.

In relation to POP outcome, no differences in fecal microbiota were observed in the current study, regardless of PS. The lack of differences could be explained by the concept that fecal microbiota is influenced by the diet and is often considered a relatively stable environment, with microbes having more functional redundancy. The direct comparison between the fecal and vaginal microbial communities in the current study indicates the fecal microbiome is a more diverse community. Therefore, the vaginal microbiota may potentially be more susceptible to dysbiosis due to the low diversity of this community, warranting additional investigation. Identifying shared OTUs between the fecal and vaginal communities may provide novel insights into any relationship between the microbiota at different body sites.

Being able to compare vaginal and fecal microbiota across the same cohort of sows presents a unique opportunity to evaluate the potential relationship between these communities. In contrast to the fecal microbiota which has direct interaction with nutrients from feed, the vaginal community is potentially influenced more by host-secretions which could explain differences in the microbiota between POP outcomes observed in previous studies (Kiefer et al., 2021b). Interestingly, many of the OTUs were observed to be present in both the fecal and vaginal communities. To our knowledge this is the first published work comparing the microbiota from different body sites in late gestation sows. Studies in other species, such as birds, have observed similar results with overlapping OTUs in various body sites, but at different abundances (Leclaire et al., 2023). Similar trends have been observed in the fecal microbiota and vaginal microbiota of sows at high risk for POP. Interestingly, the two OTUs (*Anaerococcus* and *Ezakiella*) with increased abundance in the fecal microbiota of high risk sows were decreased in vaginal microbiota of sows at high risk for POP in previous studies (Kiefer et al., 2021a,b). Some genera of interest with similar trends in high-risk sows include *Clostridium*, *Treponema*, and *Streptococcus*, all of which have significant co-occurrence networks between fecal and vaginal microbiota in the current study. All three genera have previously been linked with POP (Kiefer et al., 2021a,b) as well as other reproductive disorders (Sykes and Kalan, 1975; Rodrigues et al., 2015) and inflammation (Hughes et al., 1975). However, many of the correlations are within body sites and not between.

Investigation into the potential relationship between the vaginal and fecal communities may provide unique opportunities for POP mitigation strategies and sow reproductive health. Creating co-occurrence networks has become a popular tool for evaluating and comparing microbial populations (Williams et al., 2014; Kim et al., 2015; Cardona et al., 2016). Understanding the correlations between these communities may aid in developing prevention strategies and identifying targets for combatting POP, and the comparison of these communities provides a novel assessment of late gestation sow microbiota.

## Conclusion

Collectively, this study further validates the phenotypic perineal scoring system to identify sows at higher risk for POP. Microbial candidates of interest were identified within the fecal communities, in addition to providing further characterization of fecal and vaginal microbial populations of gestating sows. Co-occurrence networks were created between the vaginal and fecal communities providing a novel evaluation of correlations between these microbial populations, demonstrating further research is needed to better understand this interaction. Finally, this work improves the understanding of biological associations with POP in the U.S. commercial swine herd.

## Data availability statement

The datasets presented in this study can be found in the NCBI Sequence Read Archive and are available under the BioProject ID PRJNA1074313.

## Ethics statement

The animal study was approved by the Institutional Animal Care and Use Committee at Iowa State University. The study was conducted in accordance with the local legislation and institutional requirements.

## Author contributions

ZK: Conceptualization, Supervision, Data curation, Formal analysis, Investigation, Methodology, Project administration, Validation, Writing – original draft. LK: Formal analysis, Methodology, Writing – review & editing. JS: Writing – review & editing, Data curation, Project administration. SS-E: Writing – review & editing, Conceptualization, Supervision. JR: Conceptualization, Supervision, Writing – review & editing, Funding acquisition.

## References

[ref1] Acharya K. D. Gao X. Bless E. P. Chen J. Tetel M. J. (2019). Estradiol and high fat diet associate with changes in gut microbiota in female *ob*/*ob* mice. Sci. Rep. 9:20192. doi: 10.1038/s41598-019-56723-1, PMID: 31882890 PMC6934844

[ref2] Adlercreutz H. Pulkkinen M. O. Hämäläinen E. K. Korpela J. T. (1984). Studies on the role of intestinal bacteria in metabolism of synthetic and natural steroid hormones. J. Steroid Biochem. 20, 217–229. doi: 10.1016/0022-4731(84)90208-5, PMID: 6231418

[ref3] Altschul S. F. Gish W. Miller W. Myers E. W. Lipman D. J. (1990). Basic local alignment search tool. J. Mol. Biol. 215, 403–410. doi: 10.1016/S0022-2836(05)80360-2 2231712

[ref4] Anderson M. J. Willis T. J. (2003). Canonical analysis of principal coordinates: a useful method of constrained ordination for ecology. Ecology 84, 511–525. doi: 10.1890/0012-9658(2003)084[0511:CAOPCA]2.0.CO;2

[ref5] Baker J. M. Al-Nakkash L. Herbst-Kralovetz M. M. (2017). Estrogen-gut microbiome axis: physiological and clinical implications. Maturitas 103, 45–53. doi: 10.1016/j.maturitas.2017.06.025, PMID: 28778332

[ref6] Bassis C. M. Moore N. M. Lolans K. Seekatz A. M. Weinstein R. A. Young V. B.. (2017). Comparison of stool versus rectal swab samples and storage conditions on bacterial community profiles. BMC Microbiol. 17:78. doi: 10.1186/s12866-017-0983-9, PMID: 28359329 PMC5374586

[ref7] Bray J. R. Curtis J. T. (1957). An ordination of the upland forest communities of southern Wisconsin. Ecol. Monogr. 27, 325–349. doi: 10.2307/1942268

[ref8] Cardona C. Weisenhorn P. Henry C. Gilbert J. A. (2016). Network-based metabolic analysis and microbial community modeling. Curr. Opin. Microbiol. 31, 124–131. doi: 10.1016/j.mib.2016.03.008, PMID: 27060776

[ref9] Cheng C. Wei H. Yu H. Xu C. Jiang S. Peng J. (2018). Metabolic syndrome during perinatal period in sows and the link with gut microbiota and metabolites. Front. Microbiol. 9:1989. doi: 10.3389/fmicb.2018.01989, PMID: 30197635 PMC6117386

[ref10] Cotton S. Clayton C. A. Tropini C. (2023). Microbial endocrinology: the mechanisms by which the microbiota influences host sex steroids. Trends Microbiol. 31, 1131–1142. doi: 10.1016/J.TIM.2023.03.010, PMID: 37100633

[ref11] Deng F. McClure M. Rorie R. Wang X. Chai J. Wei X.. (2019). The vaginal and fecal microbiomes are related to pregnancy status in beef heifers. J. Anim. Sci. Biotechnol. 10:92. doi: 10.1186/s40104-019-0401-2, PMID: 31857897 PMC6909518

[ref12] Duncan S. H. Lobley G. E. Holtrop G. Ince J. Johnstone A. M. Louis P.. (2008). Human colonic microbiota associated with diet, obesity and weight loss. Int. J. Obes. 32, 1720–1724. doi: 10.1038/ijo.2008.155, PMID: 18779823

[ref13] Faust K. Raes J. (2016). CoNet app: inference of biological association networks using Cytoscape. F1000Res 5:1519. doi: 10.12688/f1000research.9050.2 27853510 PMC5089131

[ref14] Flores R. Shi J. Fuhrman B. Xu X. Veenstra T. D. Gail M. H.. (2012). Fecal microbial determinants of fecal and systemic estrogens and estrogen metabolites: a cross-sectional study. J. Transl. Med. 10:253. doi: 10.1186/1479-5876-10-253, PMID: 23259758 PMC3552825

[ref15] Gu Y. Zhou G. Zhou F. Li Y. Wu Q. He H.. (2022). Gut and vaginal microbiomes in PCOS: implications for Women’s health. Front. Endocrinol. 13:808508. doi: 10.3389/fendo.2022.808508, PMID: 35282446 PMC8905243

[ref16] He J. Zheng W. Tao C. Guo H. Xue Y. Zhao R.. (2020). Heat stress during late gestation disrupts maternal microbial transmission with altered offspring’s gut microbial colonization and serum metabolites in a pig model. Environ. Pollut. 266:115111. doi: 10.1016/j.envpol.2020.115111, PMID: 32663631

[ref17] Hermann-Bank M. L. Skovgaard K. Stockmarr A. Strube M. L. Larsen N. Kongsted H.. (2015). Characterization of the bacterial gut microbiota of piglets suffering from new neonatal porcine diarrhoea. BMC Vet. Res. 11:139. doi: 10.1186/s12917-015-0419-4, PMID: 26099928 PMC4476181

[ref18] Hughes R. Olander H. J. Williams C. B. (1975). Swine dysentery: pathogenicity of *Treponema hyodysenteriae*. Am. J. Vet. Res. 36, 971–977, PMID: 1147363

[ref19] Jelovsek J. E. Maher C. Barber M. D. (2007). Pelvic organ prolapse. Lancet 369, 1027–1038. doi: 10.1016/S0140-6736(07)60462-0 17382829

[ref20] Kamada N. Kim Y. G. Sham H. P. Vallance B. A. Puente J. L. Martens E. C.. (2012). Regulated virulence controls the ability of a pathogen to compete with the gut microbiota. Science 336, 1325–1329. doi: 10.1126/science.1222195 22582016 PMC3439148

[ref21] Kiefer Z. E. Koester L. R. Showman L. Studer J. M. Chipman A. L. Keating A. F.. (2021a). Vaginal microbiome and serum metabolite differences in late gestation commercial sows at risk for pelvic organ prolapse. Sci. Rep. 11:6189. doi: 10.1038/s41598-021-85367-3, PMID: 33731737 PMC7969946

[ref22] Kiefer Z. E. Koester L. R. Studer J. M. Chipman A. L. Mainquist-Whigham C. Keating A. F.. (2021b). Vaginal microbiota differences associated with pelvic organ prolapse risk during late gestation in commercial sows. Biol. Reprod. 105, 1545–1561. doi: 10.1093/BIOLRE/IOAB178, PMID: 34542158 PMC8689292

[ref23] Kiefer Z. E. Studer J. M. Chipman A. L. Adur M. K. Mainquist-Whigham C. Gabler N. K.. (2021c). Circulating biomarkers associated with pelvic organ prolapse risk in late gestation sows. J. Anim. Sci. 99:skab207. doi: 10.1093/JAS/SKAB207, PMID: 34228800 PMC8378218

[ref24] Kim J. Nguyen S. G. Guevarra R. B. Lee I. Unno T. (2015). Analysis of swine fecal microbiota at various growth stages. Arch. Microbiol. 197, 753–759. doi: 10.1007/s00203-015-1108-1, PMID: 25832348

[ref25] Koh A. De Vadder F. Kovatcheva-Datchary P. Bäckhed F. (2016). From dietary fiber to host physiology: short-chain fatty acids as key bacterial metabolites. Cell 165, 1332–1345. doi: 10.1016/j.cell.2016.05.041, PMID: 27259147

[ref26] Kozich J. J. Westcott S. L. Baxter N. T. Highlander S. K. Schloss P. D. (2013). Development of a dual-index sequencing strategy and curation pipeline for analyzing amplicon sequence data on the MiSeq Illumina sequencing platform. Appl. Environ. Microbiol. 79, 5112–5120. doi: 10.1128/AEM.01043-13, PMID: 23793624 PMC3753973

[ref27] Leclaire S. Pineaux M. Blanchard P. White J. Hatch S. A. (2023). Microbiota composition and diversity of multiple body sites vary according to reproductive performance in a seabird. Mol. Ecol. 32, 2115–2133. doi: 10.1111/mec.16398, PMID: 35152516

[ref28] Liu H. Hou C. Li N. Zhang X. Zhang G. Yang F.. (2019). Microbial and metabolic alterations in gut microbiota of sows during pregnancy and lactation. FASEB J. 33, 4490–4501. doi: 10.1096/fj.201801221RR, PMID: 30653349

[ref29] Ma C. Gao Q. K. Zhang W. H. Azad M. A. K. Kong X. F. (2020). Alterations in the blood parameters and fecal microbiota and metabolites during pregnant and lactating stages in Bama mini pigs as a model. Mediat. Inflamm. 2020, 1–13. doi: 10.1155/2020/8829072, PMID: 33162832 PMC7607286

[ref30] McMurdie P. J. Holmes S. (2013). phyloseq: an R package for reproducible interactive analysis and graphics of microbiome census data. PLoS One 8:e61217. doi: 10.1371/journal.pone.0061217, PMID: 23630581 PMC3632530

[ref31] Oksanen J. Blanche F. G. Friendly M. Kindt R. Legendre P. McGlinn D.. (2020). Vegan: community ecology package. R package version 2.5-7

[ref32] Petersen C. Round J. L. (2014). Defining dysbiosis and its influence on host immunity and disease. Cell. Microbiol. 16, 1024–1033. doi: 10.1111/cmi.12308, PMID: 24798552 PMC4143175

[ref33] Rodrigues N. F. Kästle J. Coutinho T. J. D. Amorim A. T. Campos G. B. Santos V. M.. (2015). Qualitative analysis of the vaginal microbiota of healthy cattle and cattle with genital-tract disease. Genet. Mol. Res. 14, 6518–6528. doi: 10.4238/2015.June.12.4, PMID: 26125856

[ref34] Ross J. W. (2019). Identification of putative factors contributing to pelvic organ prolapse in sows (Grant # 17-224) II. Industry Summary: Iowa State University—Iowa Pork Industry Center. Available at: https://4starvets.com/wp-content/uploads/2021/08/Final-Report_NPB-project-_17-224-002.pdf

[ref35] Sanglard L. P. Schmitz-Esser S. Gray K. A. DCL L. Yeoman C. J. JCM D.. (2020b). Vaginal microbiota diverges in sows with low and high reproductive performance after porcine reproductive and respiratory syndrome vaccination. Sci. Rep. 10, 304–306. doi: 10.1038/s41598-020-59955-8 32080317 PMC7033195

[ref36] Sanglard L. P. Schmitz-Esser S. Gray K. A. Linhares D. C. L. Yeoman C. J. Dekkers J. C. M.. (2020a). Investigating the relationship between vaginal microbiota and host genetics and their impact on immune response and farrowing traits in commercial gilts. J. Anim. Breed. Genet. 137, 84–102. doi: 10.1111/jbg.12456, PMID: 31762123

[ref37] Supakorn C. Stock J. D. Hostetler C. Stalder K. J. (2014). Prolapse incidence in swine breeding herds is a cause for concern. Open J. Vet. Med. 7, 85–97. doi: 10.4236/ojvm.2017.78009

[ref38] Sykes J. A. Kalan J. (1975). Intracellular *Treponema pallidum* in cells of a syphilitic lesion of the uterine cervix. Am. J. Obstet. Gynecol. 122, 361–367. doi: 10.1016/0002-9378(75)90185-4, PMID: 165724

[ref39] Tedjo D. I. Jonkers D. M. A. E. Savelkoul P. H. Masclee A. A. van Best N. Pierik M. J.. (2015). The effect of sampling and storage on the Fecal microbiota composition in healthy and diseased subjects. PLoS One 10:e0126685. doi: 10.1371/journal.pone.0126685, PMID: 26024217 PMC4449036

[ref40] Wang J.-F. Fu S. P. Li S. N. Hu Z. M. Xue W. J. Li Z. Q.. (2013). Short-chain fatty acids inhibit growth hormone and prolactin gene transcription via cAMP/PKA/CREB signaling pathway in dairy cow anterior pituitary cells. Int. J. Mol. Sci. 14, 21474–21488. doi: 10.3390/ijms141121474, PMID: 24177567 PMC3856016

[ref41] Wang H. Hu C. Cheng C. Cui J. Ji Y. Hao X.. (2019). Unraveling the association of fecal microbiota and oxidative stress with stillbirth rate of sows. Theriogenology 136, 131–137. doi: 10.1016/j.theriogenology.2019.06.028, PMID: 31255919

[ref42] Wang H. Ji Y. Yin C. Deng M. Tang T. Deng B.. (2018). Differential analysis of gut microbiota correlated with oxidative stress in sows with high or low litter performance during lactation. Front. Microbiol. 9:1665. doi: 10.3389/fmicb.2018.01665 30154758 PMC6103269

[ref43] Wang J. Li C. Nesengani L. T. Gong Y. Zhang S. Lu W. (2017). Characterization of vaginal microbiota of endometritis and healthy sows using high-throughput pyrosequencing of 16S rRNA gene. Microb. Pathog. 111, 325–330. doi: 10.1016/j.micpath.2017.08.030, PMID: 28867636

[ref44] Williams R. J. Howe A. Hofmockel K. S. (2014). Demonstrating microbial co-occurrence pattern analyses within and between ecosystems. Front. Microbiol. 5:358. doi: 10.3389/fmicb.2014.00358 25101065 PMC4102878

[ref45] Xu S. Dong Y. Shi J. Li Z. Che L. Lin Y.. (2021). Responses of vaginal microbiota to dietary supplementation with lysozyme and its relationship with rectal microbiota and sow performance from late gestation to early lactation. Animals 11, 1–16. doi: 10.3390/ani11030593 PMC7996156 33668266

[ref46] Yang H. Yang M. Fang S. Huang X. He M. Ke S.. (2018). Evaluating the profound effect of gut microbiome on host appetite in pigs. BMC Microbiol. 18:215. doi: 10.1186/s12866-018-1364-8, PMID: 30547751 PMC6295093

[ref47] Yuliaxis R.-C. Mach N. Lepage P. Levenez F. Denis C. Lemonnier G.. (2016). Phylogenetic network analysis applied to pig gut microbiota identifies an ecosystem structure linked with growth traits. ISME J. 10, 2973–2977. doi: 10.1038/ismej.2016.77 27177190 PMC5148198

[ref48] Zhang J. Liu M. Ke S. Huang X. Fang S. He M.. (2021). Gut and vagina microbiota associated with estrus return of weaning sows and its correlation with the changes in serum metabolites. Front. Microbiol. 12:690091. doi: 10.3389/fmicb.2021.690091 34489885 PMC8417050

[ref49] Zhong W. Fu H. Zhou Y. Yan M. Chen D. Yang M.. (2021). Identification of the gut microbiota biomarkers associated with heat cycle and failure to enter oestrus in gilts. Microb. Biotechnol. 14, 1316–1330. doi: 10.1111/1751-7915.13695 33305898 PMC8313273

