# Supplementary material for: Evaluation of fecal microbiota of late gestation sows in relation to pelvic organ prolapse risk
Source: Front Microbiol. 2024 Jul 24;15:1384583. doi: 10.3389/fmicb.2024.1384583 (PMC11303877; doi:10.3389/fmicb.2024.1384583)
Supplement: Supplementary file 1 [file Data_Sheet_1.docx]

| **Supplemental Table 1. The 50 most abundant microbes in fecal samples from sows.** | | | | | | | | | | |  |
| --- | --- | --- | --- | --- | --- | --- | --- | --- | --- | --- | --- |
|  |  |  | |  | | **NCBI BLAST** | | | | |  |
| **OTU^1^** | **Relative Abundance^2^** | | **Phylum** | | **Taxonomy (Silva v138)^3^** | | **Classification** | | **Similarity(%)** | **Accession** |  |
| OTU 1 | 5.98 | | *Turicibacter* | | *Erysipelotrichaceae* | | *Turicibacter* sp. H121 | | 99.6 | CP013476.1 | |
| OTU 2 | 5.62 | | *Clostridium_sensu_stricto_1* | | *Clostridiaceae* | | *Clostridium* sp. | | 98.0 | LC515613.1 | |
| OTU 3 | 5.19 | | *Clostridium_sensu_stricto_1* | | *Clostridiaceae* | | *Clostridium moniliforme* | | 99.6 | KY079341.1 | |
| OTU 4 | 4.97 | | *Romboutsia* | | *Peptostreptococcaceae* | | *Romboutsia* sp. CE17 | | 98.0 | CP051144.1 | |
| OTU 5 | 4.11 | | *Clostridium_sensu_stricto_1* | | *Clostridiaceae* | | *Clostridium celatum* | | 100 | LC515638.1 | |
| OTU 6 | 2.87 | | *Terrisporobacter* | | *Peptostreptococcaceae* | | *Terrisporobacter mayombei* | | 100 | MT903116.1 | |
| OTU 7 | 2.48 | | *Anaerococcus* | | *Peptostreptococcales-Tissierellales_fa* | | *Anaerococcus tetradius* | | 100 | LC036320.1 | |
| OTU 8 | 1.10 | | *Anaerococcus* | | *Peptostreptococcales-Tissierellales_fa* | | *Anaerococcus nagyae* | | 95.2 | NR_146835.1 | |
| OTU 9 | 1.67 | | *Escherichia-Shigella* | | *Enterobacteriaceae* | | *Escherichia coli* | | 100 | CP080001.1 | |
| OTU 10 | 1.36 | | *Methanobrevibacter* | | *Methanobacteriaceae* | | *Methanobrevibacter* sp. YE315 | | 100 | CP010834.1 | |
| OTU 11 | 1.75 | | *Actinomycetaceae_unclassified* | | *Actinomycetaceae* | | *Arcanobacterium hippocoleae* | | 94.5 | MH796227.1 | |
| OTU 12 | 1.33 | | *Terrisporobacter* | | *Peptostreptococcaceae* | | *Terrisporobacter* sp. CCK3R4-PYG-107 | | 99.2 | KR364793.1 | |
| OTU 13 | 1.49 | | *Anaerococcus* | | *Peptostreptococcales-Tissierellales_fa* | | *Anaerococcus sp.* | | 99.6 | MK424034.1 | |
| OTU 14 | 1.47 | | *Campylobacter* | | *Campylobacteraceae* | | *Campylobacter jejuni* | | 100 | CP048760.1 | |
| OTU 15 | 1.14 | | *Clostridium_sensu_stricto_1* | | *Clostridiaceae* | | *Clostridium butyricum* | | 100 | MT510294.1 | |
| OTU 16 | 1.10 | | *Clostridium_sensu_stricto_1* | | *Clostridiaceae* | | *Clostridium sp.* | | 100 | MN227345.1 | |
| OTU 17 | 0.87 | | *Streptococcus* | | *Streptococcaceae* | | *Streptococcus alactolyticus* | | 100 | MT597633.1 | |
| OTU 18 | 1.31 | | *Bacteroides* | | *Bacteroidaceae* | | *Bacteroides fragilis* | | 99.6 | CP054003.1 | |
| OTU 19 | 0.93 | | *Fusobacterium* | | *Fusobacteriaceae* | | *Fusobacterium gastrosuis* | | 99.6 | LT607734.1 | |
| OTU 20 | 1.01 | | *Treponema* | | *Spirochaetaceae* | | *Treponema bryantii* | | 99.6 | AB849328.1 | |
| OTU 21 | 0.74 | | *Christensenellaceae* | | *Christensenellaceae* | | *Clostridiales* *bacterium* | | 99.6 | JN713556.1 | |
| OTU 22 | 0.54 | | *Prevotellaceae_unclassified* | | *Prevotellaceae* | | *Rumen bacterium* NK3B31 | | 89.9 | GU324411.1 | |
| OTU 23 | 1.20 | | *Corynebacterium* | | *Corynebacteriaceae* | | *Corynebacterium amycolatum* | | 99.6 | MT423433.1 | |
| OTU 24 | 0.67 | | *Fenollaria* | | *Peptostreptococcales-Tissierellales_fa* | | *Peptostreptococcaceae bacterium* | | 99.6 | CP027242.1 | |
| OTU 25 | 0.84 | | *Prevotellaceae_UCG-001* | | *Prevotellaceae* | | *Duncaniella freteri* | | 84.7 | MK521456.1 | |
| OTU 26 | 0.86 | | *Bacteroides* | | *Bacteroidaceae* | | *Phocaeicola vulgatus* | | 100 | MT515977.1 | |
| OTU 27 | 0.67 | | *Kurthia* | | *Planococcaceae* | | *Rummeliibacillus sp.* | | 99.6 | MK053894.1 | |
| OTU 28 | 0.73 | | *Porphyromonas* | | *Porphyromonadaceae* | | *Porphyromonas katsikii* | | 92.1 | KM360064.1 | |
| OTU 29 | 0.94 | | *Prevotellaceae_unclassified* | | *Prevotellaceae* | | *Prevotella buccalis* | | 99.6 | JN867261.1 | |
| OTU 30 | 0.73 | | *Corynebacterium* | | *Corynebacteriaceae* | | *Corynebacterium xerosis* | | 99.6 | MT482630.1 | |
| OTU 31 | 0.62 | | *Peptoniphilus* | | *Peptostreptococcales-Tissierellales_fa* | | *Peptoniphilus faecalis* | | 99.6 | MT396160.1 | |
| OTU 32 | 0.67 | | *p-251-o5_ge(98)* | | *p-251-o5(98)* | | *Muribaculum sp.* | | 85.4 | MK287698.1 | |
| OTU 33 | 0.64 | | *Lactobacillus* | | *Lactobacillaceae* | | *Lactobacillus amylovorus* | | 100 | MT459395.1 | |
| OTU 34 | 0.56 | | *Finegoldia* | | *Peptostreptococcales-Tissierellales_fa* | | *Finegoldia magna* | | 99.6 | CP054000.1 | |
| OTU 35 | 0.41 | | *Clostridium_sensu_stricto_1* | | *Clostridiaceae* | | *Clostridium cellulovorans* | | 100 | KF528156.1 | |
| OTU 36 | 0.27 | | *Bacteria_unclassified* | | *Bacteria_unclassified* | | *Anaerococcus* sp. Marseille-P9784 | | 88.9 | LR745663.1 | |
| OTU 37 | 0.29 | | *Nosocomiicoccus* | | *Staphylococcaceae* | | *Nosocomiicoccus ampullae* | | 99.6 | CP079109.1 | |
| OTU 38 | 0.64 | | *Actinobacillus* | | *Pasteurellaceae* | | *Terrahaemophilus aromaticivorans* | | 99.6 | KC632195.1 | |
| OTU 39 | 0.46 | | *Ruminococcus* | | *Ruminococcaceae* | | *Ruminococcus flavefaciens* | | 98.8 | AF104841.1 | |
| OTU 40 | 0.40 | | *Firmicutes_unclassified* | | *Firmicutes_unclassified* | | *Bacterium* | | 92.5 | LC378703.1 | |
| OTU 41 | 0.38 | | *Actinomycetaceae_unclassified* | | *Actinomycetaceae* | | *Actinomyces faecalis* | | 95.7 | MT256397.1 | |
| OTU 42 | 0.45 | | *Porphyromonas* | | *Porphyromonadaceae* | | *Porphyromonas somerae* | | 99.6 | KP192301.1 | |
| OTU 43 | 0.36 | | *Lachnospiraceae_XPB1014_group* | | *Lachnospiraceae* | | *Lachnospiraceae bacterium* CA63 | | 94.5 | AB849414.1 | |
| OTU 44 | 0.35 | | *Peptostreptococcus* | | *Peptostreptococcaceae* | | *Peptostreptococcus porci* | | 99.6 | MN537513.1 | |
| OTU 45 | 0.40 | | *Bacteroidales_unclassified* | | *Bacteroidales_unclassified* | | *Muribaculum sp.* | | 85.4 | MK287698.1 | |
| OTU 46 | 0.12 | | *Ezakiella* | | *Peptostreptococcales-Tissierellales_fa* | | *Ezakiella coagulans* | | 97.6 | NR_104900.1 | |
| OTU 47 | 0.30 | | *Bacteroides* | | *Bacteroidaceae* | | *Phocaeicola plebeius* | | 99.6 | MT749279.1 | |
| OTU 48 | 0.30 | | *Lachnospiraceae_UCG-007* | | *Lachnospiraceae* | | *Lachnotalea glycerini* | | 97.2 | MF953294.1 | |
| OTU 49 | 0.23 | | *Anaerococcus* | | *Peptostreptococcales-Tissierellales_fa* | | *Anaerococcus provencensis* | | 94.5 | NR_133036.1 | |
| OTU 50 | 0.16 | | *Bacteroides* | | *Bacteroidaceae* | | *Phocaeicola massiliensis* | | 96.1 | LC515611.1 | |
| ^1^ Individual microbes were assigned in order of abundance and classified into operational taxonomic units (OTUs).  ^2^ Relative abundance of the specific OTU in the fecal swabs collected in this study. Represented as a percent.  ^3^ Taxonomy was assigned using Silva SSU NR reference database (v138). | | | | | | | |  |  |  |  |

**
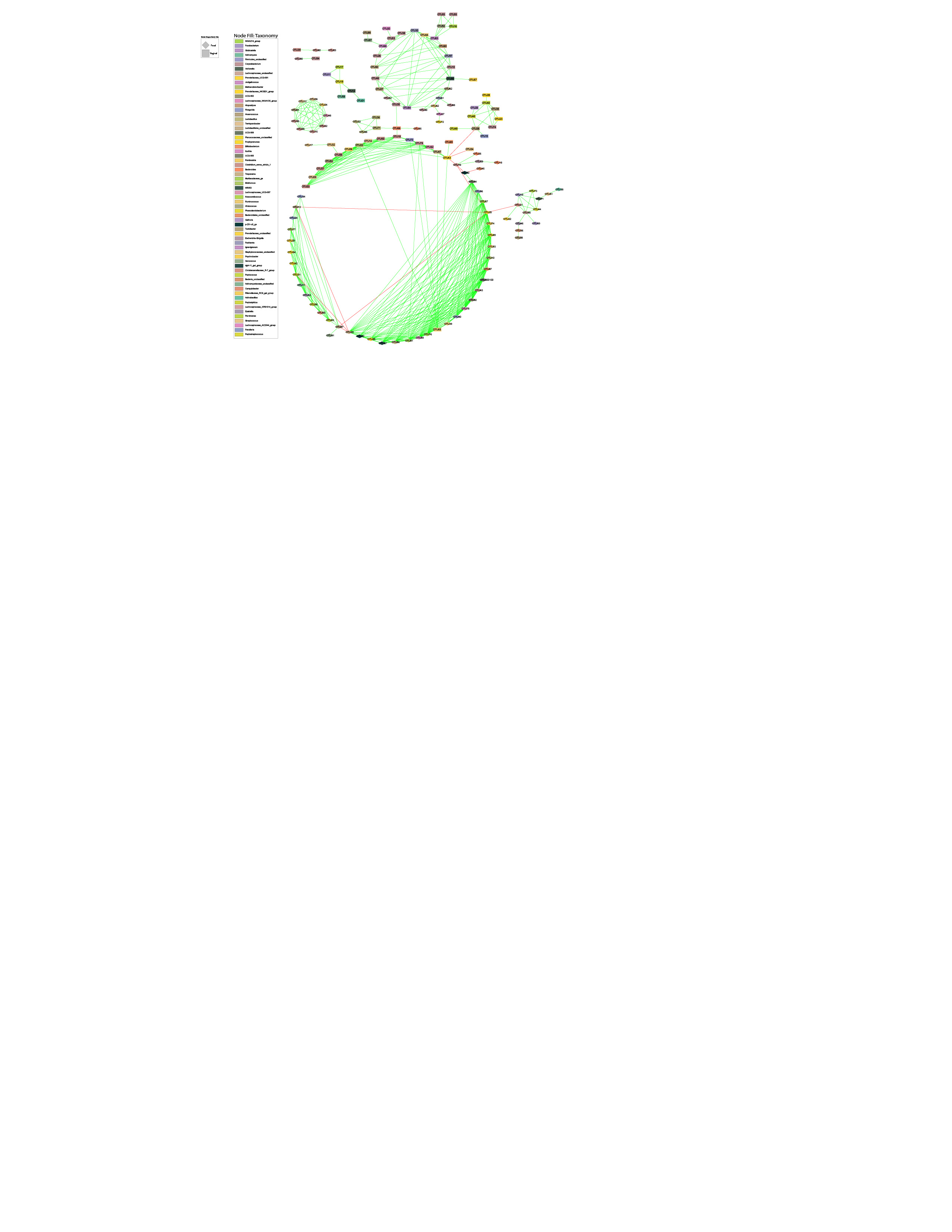
**

**Supplementary Figure 1. Co-Occurrence network comparing the fecal and vaginal microbiota of late gestation sows.**

Co-Occurrence network created using the CoNet (v 1.1.1) application with Cytoscape showing the significant (*P* < 0.05) correlations between the top 100 most abundant OTU’s from the fecal and vaginal microbiota of late gestation sows. Nodes represent each individual OTU, and the color representing taxonomy. Edges in green are positive correlations, while those in red are negative.

| **Supplementary Table 2. Edge scores connecting the fecal and vaginal nodes^1^** | | | | |
| --- | --- | --- | --- | --- |
| **Label^2^** | ***P -*Value** | | | |
|  | **Pearson^3^** | **Spearman^4^** | **Bray Curtis^5^** | **Kullback-Leibler^6^** |
| OTU 1 Fecal->OTU 5 Fecal | < 0.01 | < 0.01 | 0.04 | < 0.01 |
| OTU 1 Fecal->OTU 6 Fecal | < 0.01 | < 0.01 | 0.17 | 0.02 |
| OTU 1 Fecal->OTU 12 Fecal | < 0.01 | < 0.01 | 0.06 | 0.01 |
| OTU 1 Fecal->OTU 35 Fecal | < 0.01 | < 0.01 | 0.09 | 0.01 |
| OTU 1 Fecal->OTU 48 Fecal | < 0.01 | < 0.01 | 0.16 | 0.01 |
| OTU 2 Fecal->OTU 1 Fecal | < 0.01 | < 0.01 | 0.04 | < 0.01 |
| OTU 2 Fecal->OTU 4 Fecal | 0.01 | < 0.01 | 0.11 | 0.07 |
| OTU 2 Fecal->OTU 5 Fecal | < 0.01 | < 0.01 | 0.02 | < 0.01 |
| OTU 2 Fecal->OTU 6 Fecal | < 0.01 | < 0.01 | 0.05 | 0.01 |
| OTU 2 Fecal->OTU 12 Fecal | < 0.01 | < 0.01 | 0.07 | 0.03 |
| OTU 2 Fecal->OTU 15 Fecal | 0.02 | < 0.01 | 0.16 | 0.06 |
| OTU 2 Fecal->OTU 35 Fecal | < 0.01 | < 0.01 | 0.10 | 0.01 |
| OTU 2 Fecal->OTU 48 Fecal | < 0.01 | < 0.01 | 0.07 | < 0.01 |
| OTU 2 Vaginal->OTU 3 Vaginal | < 0.01 | < 0.01 | 0.01 | < 0.01 |
| OTU 3 Fecal->OTU 60 Fecal | 0.02 | < 0.01 | 0.12 | < 0.01 |
| OTU 4 Fecal->OTU 1 Fecal | < 0.01 | < 0.01 | 0.07 | 0.03 |
| OTU 4 Fecal->OTU 5 Fecal | < 0.01 | < 0.01 | 0.09 | 0.01 |
| OTU 4 Fecal->OTU 6 Fecal | < 0.01 | < 0.01 | 0.09 | < 0.01 |
| OTU 4 Fecal->OTU 12 Fecal | < 0.01 | < 0.01 | 0.04 | < 0.01 |
| OTU 4 Fecal->OTU 35 Fecal | < 0.01 | < 0.01 | 0.05 | < 0.01 |
| OTU 4 Fecal->OTU 48 Fecal | < 0.01 | < 0.01 | 0.16 | 0.03 |
| OTU 5 Fecal->OTU 6 Fecal | < 0.01 | < 0.01 | 0.10 | < 0.01 |
| OTU 5 Fecal->OTU 12 Fecal | < 0.01 | < 0.01 | 0.03 | < 0.01 |
| OTU 5 Fecal->OTU 15 Fecal | 0.04 | < 0.01 | 0.21 | 0.08 |
| OTU 5 Fecal->OTU 35 Fecal | < 0.01 | < 0.01 | 0.02 | < 0.01 |
| OTU 5 Fecal->OTU 48 Fecal | < 0.01 | < 0.01 | 0.09 | < 0.01 |
| OTU 5 Vaginal->OTU 2 Vaginal | 0.01 | < 0.01 | 0.18 | 0.01 |
| OTU 5 Vaginal->OTU 3 Vaginal | 0.03 | 0.01 | 0.21 | 0.02 |
| OTU 5 Vaginal->OTU 7 Vaginal | 0.03 | < 0.01 | 0.20 | 0.01 |
| OTU 5 Vaginal->OTU 32 Vaginal | 0.07 | 0.01 | 0.18 | 0.01 |
| OTU 6 Fecal->OTU 35 Fecal | < 0.01 | < 0.01 | 0.12 | < 0.01 |
| OTU 6 Fecal->OTU 48 Fecal | < 0.01 | < 0.01 | 0.11 | 0.01 |
| OTU 6 Vaginal->OTU 2 Vaginal | < 0.01 | < 0.01 | 0.01 | < 0.01 |
| OTU 6 Vaginal->OTU 3 Vaginal | < 0.01 | < 0.01 | 0.01 | < 0.01 |
| OTU 6 Vaginal->OTU 5 Vaginal | 0.01 | < 0.01 | 0.17 | < 0.01 |
| OTU 6 Vaginal->OTU 7 Vaginal | < 0.01 | < 0.01 | 0.01 | < 0.01 |
| OTU 6 Vaginal->OTU 9 Vaginal | < 0.01 | < 0.01 | 0.02 | < 0.01 |
| OTU 7 Vaginal->OTU 2 Vaginal | < 0.01 | < 0.01 | 0.01 | < 0.01 |
| OTU 7 Vaginal->OTU 3 Vaginal | < 0.01 | < 0.01 | < 0.01 | < 0.01 |
| OTU 7 Vaginal->OTU 9 Vaginal | < 0.01 | < 0.01 | 0.02 | < 0.01 |
| OTU 8 Vaginal->OTU 34 Vaginal | 0.04 | < 0.01 | 0.07 | < 0.01 |
| OTU 8 Vaginal->OTU 49 Vaginal | 0.01 | 0.01 | 0.05 | < 0.01 |
| OTU 9 Fecal->OTU 18 Fecal | 0.05 | < 0.01 | . | . |
| OTU 9 Vaginal->OTU 2 Vaginal | < 0.01 | < 0.01 | 0.06 | < 0.01 |
| OTU 9 Vaginal->OTU 3 Vaginal | < 0.01 | < 0.01 | < 0.01 | < 0.01 |
| OTU 10 Fecal->OTU 43 Fecal | 0.05 | < 0.01 | 0.11 | < 0.01 |
| OTU 10 Fecal->OTU 57 Fecal | 0.06 | < 0.01 | 0.15 | 0.01 |
| OTU 10 Fecal->OTU 64 Fecal | 0.05 | < 0.01 | 0.20 | < 0.01 |
| OTU 10 Fecal->OTU 68 Fecal | 0.06 | < 0.01 | 0.12 | 0.02 |
| OTU 10 Fecal->OTU 76 Fecal | 0.03 | < 0.01 | 0.15 | < 0.01 |
| OTU 10 Fecal->OTU 81 Fecal | 0.03 | < 0.01 | 0.18 | < 0.01 |
| OTU 10 Fecal->OTU 89 Fecal | 0.10 | 0.01 | 0.21 | 0.01 |
| OTU 10 Vaginal->OTU 64 Vaginal | < 0.01 | 0.01 | 0.04 | < 0.01 |
| OTU 10 Vaginal->OTU 88 Vaginal | 0.01 | < 0.01 | 0.03 | < 0.01 |
| OTU 10 Vaginal->OTU 97 Vaginal | 0.02 | 0.01 | 0.05 | < 0.01 |
| OTU 11 Vaginal->OTU 17 Vaginal | 0.15 | < 0.01 | 0.09 | . |
| OTU 11 Vaginal->OTU 19 Vaginal | 0.12 | < 0.01 | 0.07 | . |
| OTU 12 Fecal->OTU 6 Fecal | < 0.01 | < 0.01 | 0.08 | < 0.01 |
| OTU 12 Fecal->OTU 35 Fecal | < 0.01 | < 0.01 | 0.05 | < 0.01 |
| OTU 12 Fecal->OTU 48 Fecal | < 0.01 | < 0.01 | 0.14 | 0.01 |
| OTU 12 Vaginal->OTU 1 Vaginal | 0.15 | < 0.01 | 0.09 | . |
| OTU 12 Vaginal->OTU 19 Vaginal | 0.10 | < 0.01 | 0.06 | < 0.01 |
| OTU 13 Fecal->OTU 7 Fecal | 0.03 | < 0.01 | 0.09 | < 0.01 |
| OTU 13 Fecal->OTU 24 Fecal | 0.08 | < 0.01 | . | . |
| OTU 13 Fecal->OTU 25 Fecal | 0.05 | 0.05 | . | < 0.01 |
| OTU 13 Fecal->OTU 29 Fecal | 0.04 | < 0.01 | 0.08 | . |
| OTU 13 Fecal->OTU 31 Fecal | < 0.01 | < 0.01 | 0.03 | < 0.01 |
| OTU 13 Fecal->OTU 34 Fecal | 0.03 | < 0.01 | 0.07 | < 0.01 |
| OTU 13 Fecal->OTU 56 Fecal | 0.01 | < 0.01 | 0.03 | < 0.01 |
| OTU 14 Vaginal->OTU 2 Vaginal | < 0.01 | < 0.01 | 0.01 | < 0.01 |
| OTU 14 Vaginal->OTU 3 Vaginal | < 0.01 | < 0.01 | 0.01 | < 0.01 |
| OTU 14 Vaginal->OTU 5 Vaginal | 0.02 | 0.01 | 0.19 | 0.01 |
| OTU 14 Vaginal->OTU 6 Vaginal | < 0.01 | < 0.01 | < 0.01 | < 0.01 |
| OTU 14 Vaginal->OTU 7 Vaginal | < 0.01 | < 0.01 | < 0.01 | < 0.01 |
| OTU 14 Vaginal->OTU 9 Vaginal | < 0.01 | < 0.01 | 0.02 | < 0.01 |
| OTU 14 Vaginal->OTU 50 Vaginal | < 0.01 | < 0.01 | 0.01 | < 0.01 |
| OTU 15 Fecal->OTU 6 Fecal | 0.03 | < 0.01 | 0.16 | 0.04 |
| OTU 15 Fecal->OTU 12 Fecal | 0.03 | < 0.01 | 0.21 | 0.06 |
| OTU 15 Fecal->OTU 48 Fecal | 0.09 | < 0.01 | 0.17 | 0.08 |
| OTU 15 Vaginal->OTU 8 Vaginal | < 0.01 | < 0.01 | 0.03 | < 0.01 |
| OTU 16 Fecal->OTU 9 Fecal | 0.06 | 0.01 | 0.11 | < 0.01 |
| OTU 16 Fecal->OTU 25 Fecal | 0.05 | 0.05 | . | < 0.01 |
| OTU 16 Fecal->OTU 26 Fecal | 0.03 | 0.01 | . | . |
| OTU 16 Fecal->OTU 43 Vaginal | . | 0.06 | . | < 0.01 |
| OTU 16 Vaginal->OTU 83 Vaginal | 0.01 | < 0.01 | 0.05 | < 0.01 |
| OTU 16 Vaginal->OTU 92 Vaginal | < 0.01 | < 0.01 | < 0.01 | < 0.01 |
| OTU 16 Vaginal->OTU 93 Vaginal | 0.01 | 0.01 | 0.04 | < 0.01 |
| OTU 17 Fecal->OTU 32 Vaginal | 0.02 | < 0.01 | 0.08 | < 0.01 |
| OTU 18 Vaginal->OTU 2 Vaginal | 0.11 | < 0.01 | 0.15 | 0.04 |
| OTU 18 Vaginal->OTU 3 Vaginal | < 0.01 | < 0.01 | 0.06 | < 0.01 |
| OTU 18 Vaginal->OTU 6 Vaginal | 0.06 | < 0.01 | 0.12 | 0.01 |
| OTU 18 Vaginal->OTU 7 Vaginal | 0.02 | < 0.01 | 0.09 | < 0.01 |
| OTU 18 Vaginal->OTU 9 Vaginal | 0.02 | < 0.01 | 0.09 | < 0.01 |
| OTU 18 Vaginal->OTU 14 Vaginal | 0.02 | < 0.01 | 0.10 | < 0.01 |
| OTU 18 Vaginal->OTU 50 Vaginal | 0.02 | < 0.01 | 0.10 | < 0.01 |
| OTU 18 Vaginal->OTU 66 Vaginal | . | 0.02 | 0.27 | < 0.01 |
| OTU 18 Vaginal->OTU 73 Vaginal | 0.08 | 0.01 | 0.19 | < 0.01 |
| OTU 19 Fecal->OTU 21 Fecal | 0.01 | < 0.01 | 0.04 | < 0.01 |
| OTU 19 Fecal->OTU 44 Fecal | < 0.01 | < 0.01 | < 0.01 | < 0.01 |
| OTU 19 Fecal->OTU 71 Fecal | 0.02 | < 0.01 | 0.03 | < 0.01 |
| OTU 19 Fecal->OTU 73 Fecal | 0.05 | < 0.01 | 0.08 | < 0.01 |
| OTU 19 Fecal->OTU 98 Fecal | 0.03 | 0.02 | . | < 0.01 |
| OTU 19 Vaginal->OTU 17 Vaginal | < 0.01 | < 0.01 | 0.01 | < 0.01 |
| OTU 19 Vaginal->OTU 58 Vaginal | 0.10 | < 0.01 | . | . |
| OTU 20 Fecal->OTU 7 Fecal | 0.13 | 0.06 | . | < 0.01 |
| OTU 20 Fecal->OTU 10 Fecal | . | 0.01 | . | . |
| OTU 20 Fecal->OTU 13 Fecal | 0.13 | 0.06 | 0.17 | < 0.01 |
| OTU 20 Fecal->OTU 25 Fecal | < 0.01 | < 0.01 | 0.01 | < 0.01 |
| OTU 20 Fecal->OTU 31 Fecal | 0.08 | 0.04 | . | < 0.01 |
| OTU 20 Fecal->OTU 40 Fecal | 0.07 | < 0.01 | . | . |
| OTU 20 Fecal->OTU 43 Fecal | < 0.01 | < 0.01 | 0.07 | < 0.01 |
| OTU 20 Fecal->OTU 57 Fecal | 0.02 | < 0.01 | 0.09 | < 0.01 |
| OTU 20 Fecal->OTU 64 Fecal | 0.03 | < 0.01 | 0.07 | < 0.01 |
| OTU 20 Fecal->OTU 67 Fecal | 0.01 | < 0.01 | 0.04 | < 0.01 |
| OTU 20 Fecal->OTU 68 Fecal | 0.02 | < 0.01 | 0.08 | < 0.01 |
| OTU 20 Fecal->OTU 70 Fecal | . | < 0.01 | . | . |
| OTU 20 Fecal->OTU 74 Fecal | 0.03 | < 0.01 | 0.06 | < 0.01 |
| OTU 20 Fecal->OTU 76 Fecal | 0.01 | < 0.01 | 0.11 | < 0.01 |
| OTU 20 Fecal->OTU 78 Fecal | < 0.01 | < 0.01 | 0.04 | < 0.01 |
| OTU 20 Fecal->OTU 81 Fecal | 0.03 | < 0.01 | . | . |
| OTU 20 Fecal->OTU 85 Fecal | 0.04 | < 0.01 | 0.09 | < 0.01 |
| OTU 20 Fecal->OTU 86 Fecal | 0.06 | < 0.01 | 0.08 | < 0.01 |
| OTU 20 Fecal->OTU 87 Fecal | 0.08 | < 0.01 | . | . |
| OTU 20 Fecal->OTU 89 Fecal | 0.01 | < 0.01 | 0.10 | < 0.01 |
| OTU 20 Fecal->OTU 93 Fecal | 0.06 | 0.01 | 0.11 | < 0.01 |
| OTU 21 Fecal->OTU 44 Fecal | 0.01 | < 0.01 | 0.06 | < 0.01 |
| OTU 22 Fecal->OTU 21 Fecal | < 0.01 | 0.01 | 0.04 | < 0.01 |
| OTU 22 Vaginal->OTU 2 Vaginal | 0.01 | < 0.01 | 0.12 | < 0.01 |
| OTU 22 Vaginal->OTU 3 Vaginal | < 0.01 | < 0.01 | 0.10 | < 0.01 |
| OTU 22 Vaginal->OTU 5 Vaginal | 0.03 | < 0.01 | 0.14 | < 0.01 |
| OTU 22 Vaginal->OTU 6 Vaginal | < 0.01 | < 0.01 | 0.06 | < 0.01 |
| OTU 22 Vaginal->OTU 7 Vaginal | 0.01 | < 0.01 | 0.12 | < 0.01 |
| OTU 22 Vaginal->OTU 9 Vaginal | < 0.01 | < 0.01 | 0.10 | < 0.01 |
| OTU 22 Vaginal->OTU 14 Vaginal | < 0.01 | < 0.01 | 0.09 | < 0.01 |
| OTU 22 Vaginal->OTU 18 Vaginal | 0.01 | < 0.01 | 0.11 | < 0.01 |
| OTU 22 Vaginal->OTU 43 Vaginal | 0.04 | < 0.01 | . | . |
| OTU 22 Vaginal->OTU 47 Vaginal | 0.08 | < 0.01 | . | . |
| OTU 22 Vaginal->OTU 50 Vaginal | < 0.01 | < 0.01 | 0.10 | < 0.01 |
| OTU 22 Vaginal->OTU 65 Vaginal | 0.03 | 0.01 | 0.14 | < 0.01 |
| OTU 22 Vaginal->OTU 73 Vaginal | 0.02 | < 0.01 | 0.12 | < 0.01 |
| OTU 23 Vaginal->OTU 74 Vaginal | 0.08 | 0.01 | . | < 0.01 |
| OTU 24 Fecal->OTU 31 Fecal | 0.01 | 0.02 | 0.08 | < 0.01 |
| OTU 24 Fecal->OTU 56 Fecal | < 0.01 | 0.01 | 0.06 | < 0.01 |
| OTU 25 Fecal->OTU 100 Fecal | 0.02 | < 0.01 | 0.11 | < 0.01 |
| OTU 25 Fecal->OTU 7 Fecal | 0.07 | 0.06 | . | < 0.01 |
| OTU 25 Fecal->OTU 10 Fecal | 0.05 | < 0.01 | 0.15 | < 0.01 |
| OTU 25 Fecal->OTU 21 Fecal | 0.07 | 0.04 | 0.18 | < 0.01 |
| OTU 25 Fecal->OTU 43 Fecal | < 0.01 | < 0.01 | 0.03 | < 0.01 |
| OTU 25 Fecal->OTU 57 Fecal | < 0.01 | < 0.01 | 0.02 | < 0.01 |
| OTU 25 Fecal->OTU 64 Fecal | < 0.01 | < 0.01 | 0.02 | < 0.01 |
| OTU 25 Fecal->OTU 68 Fecal | < 0.01 | < 0.01 | 0.01 | < 0.01 |
| OTU 25 Fecal->OTU 70 Fecal | 0.09 | < 0.01 | 0.08 | . |
| OTU 25 Fecal->OTU 74 Fecal | 0.02 | < 0.01 | 0.06 | < 0.01 |
| OTU 25 Fecal->OTU 76 Fecal | < 0.01 | < 0.01 | 0.06 | < 0.01 |
| OTU 25 Fecal->OTU 78 Fecal | < 0.01 | < 0.01 | 0.03 | < 0.01 |
| OTU 25 Fecal->OTU 81 Fecal | < 0.01 | < 0.01 | 0.02 | < 0.01 |
| OTU 25 Fecal->OTU 85 Fecal | < 0.01 | < 0.01 | < 0.01 | < 0.01 |
| OTU 25 Fecal->OTU 87 Fecal | 0.01 | < 0.01 | 0.06 | < 0.01 |
| OTU 25 Fecal->OTU 89 Fecal | < 0.01 | < 0.01 | 0.02 | < 0.01 |
| OTU 25 Fecal->OTU 93 Fecal | 0.02 | 0.02 | 0.13 | < 0.01 |
| OTU 26 Vaginal->OTU 38 Vaginal | 0.06 | < 0.01 | . | . |
| OTU 26 Vaginal->OTU 60 Vaginal | 0.08 | < 0.01 | 0.05 | < 0.01 |
| OTU 26 Vaginal->OTU 68 Vaginal | . | < 0.01 | . | . |
| OTU 27 Fecal->OTU 72 Fecal | 0.06 | < 0.01 | . | . |
| OTU 27 Vaginal->OTU 30 Vaginal | 0.10 | 0.01 | 0.18 | < 0.01 |
| OTU 27 Vaginal->OTU 35 Vaginal | . | 0.07 | 0.21 | < 0.01 |
| OTU 27 Vaginal->OTU 52 Fecal | 0.06 | 0.02 | 0.13 | < 0.01 |
| OTU 27 Vaginal->OTU 64 Vaginal | < 0.01 | < 0.01 | 0.02 | < 0.01 |
| OTU 27 Vaginal->OTU 88 Vaginal | 0.05 | 0.02 | 0.14 | < 0.01 |
| OTU 27 Vaginal->OTU 97 Vaginal | 0.08 | 0.03 | 0.18 | < 0.01 |
| OTU 28 Vaginal->OTU 40 Vaginal | 0.02 | 0.01 | 0.04 | < 0.01 |
| OTU 29 Fecal->OTU 31 Fecal | 0.02 | < 0.01 | 0.07 | < 0.01 |
| OTU 29 Fecal->OTU 77 Fecal | 0.07 | < 0.01 | . | . |
| OTU 30 Fecal->OTU 52 Fecal | 0.03 | 0.01 | 0.05 | < 0.01 |
| OTU 30 Fecal->OTU 82 Fecal | < 0.01 | < 0.01 | < 0.01 | < 0.01 |
| OTU 31 Fecal->OTU 7 Fecal | 0.06 | 0.01 | 0.12 | < 0.01 |
| OTU 31 Fecal->OTU 11 Fecal | 0.02 | < 0.01 | 0.06 | < 0.01 |
| OTU 31 Fecal->OTU 56 Fecal | < 0.01 | < 0.01 | 0.02 | < 0.01 |
| OTU 32 Fecal->OTU 16 Fecal | 0.12 | 0.10 | 0.30 | < 0.01 |
| OTU 32 Fecal->OTU 45 Fecal | . | 0.17 | 0.02 | < 0.01 |
| OTU 32 Fecal->OTU 64 Fecal | 0.01 | < 0.01 | . | . |
| OTU 33 Fecal->OTU 36 Vaginal | 0.01 | < 0.01 | 0.03 | < 0.01 |
| OTU 34 Fecal->OTU 7 Fecal | 0.02 | 0.01 | 0.07 | < 0.01 |
| OTU 34 Fecal->OTU 31 Fecal | 0.10 | 0.01 | 0.13 | < 0.01 |
| OTU 35 Vaginal->OTU 64 Vaginal | 0.04 | < 0.01 | 0.12 | < 0.01 |
| OTU 36 Fecal->OTU 8 Fecal | 0.02 | 0.01 | 0.08 | < 0.01 |
| OTU 38 Fecal->OTU 51 Fecal | 0.05 | < 0.01 | . | . |
| OTU 38 Vaginal->OTU 30 Vaginal | 0.02 | < 0.01 | 0.07 | < 0.01 |
| OTU 39 Fecal->OTU 40 Fecal | 0.03 | 0.01 | 0.12 | < 0.01 |
| OTU 39 Fecal->OTU 65 Vaginal | 0.01 | 0.01 | 0.05 | < 0.01 |
| OTU 39 Fecal->OTU 68 Fecal | 0.06 | 0.06 | 0.19 | 0.01 |
| OTU 39 Fecal->OTU 76 Fecal | 0.01 | 0.02 | 0.10 | < 0.01 |
| OTU 39 Vaginal->OTU 60 Fecal | 0.06 | 0.01 | 0.20 | < 0.01 |
| OTU 40 Fecal->OTU 76 Fecal | 0.01 | < 0.01 | 0.05 | < 0.01 |
| OTU 40 Fecal->OTU 93 Fecal | 0.07 | < 0.01 | 0.12 | < 0.01 |
| OTU 40 Vaginal->OTU 23 Vaginal | 0.02 | 0.01 | 0.04 | < 0.01 |
| OTU 40 Vaginal->OTU 25 Vaginal | < 0.01 | < 0.01 | < 0.01 | < 0.01 |
| OTU 40 Vaginal->OTU 34 Vaginal | 0.08 | < 0.01 | 0.05 | . |
| OTU 40 Vaginal->OTU 74 Vaginal | 0.03 | 0.01 | 0.06 | < 0.01 |
| OTU 42 Fecal->OTU 31 Fecal | 0.04 | 0.01 | 0.10 | < 0.01 |
| OTU 43 Fecal->OTU 40 Fecal | 0.10 | 0.02 | 0.23 | 0.01 |
| OTU 43 Fecal->OTU 68 Fecal | < 0.01 | < 0.01 | < 0.01 | < 0.01 |
| OTU 43 Fecal->OTU 76 Fecal | < 0.01 | < 0.01 | 0.03 | < 0.01 |
| OTU 43 Fecal->OTU 81 Fecal | 0.02 | < 0.01 | 0.06 | < 0.01 |
| OTU 43 Fecal->OTU 85 Fecal | 0.01 | < 0.01 | 0.06 | < 0.01 |
| OTU 43 Fecal->OTU 93 Fecal | 0.04 | 0.06 | 0.17 | < 0.01 |
| OTU 43 Vaginal->OTU 4 Vaginal | . | . | . | < 0.01 |
| OTU 43 Vaginal->OTU 8 Vaginal | . | 0.05 | . | < 0.01 |
| OTU 43 Vaginal->OTU 65 Vaginal | 0.02 | 0.01 | 0.06 | < 0.01 |
| OTU 43 Vaginal->OTU 73 Vaginal | 0.08 | < 0.01 | 0.11 | . |
| OTU 44 Vaginal->OTU 10 Vaginal | < 0.01 | < 0.01 | 0.02 | < 0.01 |
| OTU 44 Vaginal->OTU 30 Vaginal | 0.13 | < 0.01 | 0.14 | < 0.01 |
| OTU 44 Vaginal->OTU 64 Vaginal | 0.07 | < 0.01 | 0.10 | < 0.01 |
| OTU 44 Vaginal->OTU 88 Vaginal | 0.02 | < 0.01 | 0.06 | < 0.01 |
| OTU 44 Vaginal->OTU 97 Vaginal | 0.03 | < 0.01 | 0.04 | < 0.01 |
| OTU 45 Vaginal->OTU 8 Vaginal | < 0.01 | < 0.01 | 0.03 | < 0.01 |
| OTU 45 Vaginal->OTU 25 Vaginal | < 0.01 | 0.01 | 0.05 | . |
| OTU 45 Vaginal->OTU 34 Vaginal | 0.03 | < 0.01 | 0.04 | < 0.01 |
| OTU 45 Vaginal->OTU 40 Vaginal | 0.03 | < 0.01 | 0.03 | < 0.01 |
| OTU 45 Vaginal->OTU 74 Vaginal | 0.02 | 0.01 | 0.06 | < 0.01 |
| OTU 46 Fecal->OTU 36 Fecal | 0.01 | 0.02 | 0.06 | < 0.01 |
| OTU 46 Vaginal->OTU 10 Vaginal | < 0.01 | < 0.01 | < 0.01 | < 0.01 |
| OTU 46 Vaginal->OTU 27 Vaginal | 0.14 | 0.02 | 0.14 | < 0.01 |
| OTU 46 Vaginal->OTU 44 Vaginal | < 0.01 | < 0.01 | 0.01 | < 0.01 |
| OTU 46 Vaginal->OTU 64 Vaginal | 0.04 | < 0.01 | 0.05 | < 0.01 |
| OTU 46 Vaginal->OTU 88 Vaginal | 0.02 | < 0.01 | 0.05 | < 0.01 |
| OTU 46 Vaginal->OTU 97 Vaginal | 0.01 | 0.01 | 0.04 | < 0.01 |
| OTU 47 Vaginal->OTU 43 Vaginal | < 0.01 | < 0.01 | 0.01 | < 0.01 |
| OTU 47 Vaginal->OTU 65 Vaginal | 0.05 | < 0.01 | 0.07 | < 0.01 |
| OTU 47 Vaginal->OTU 73 Vaginal | 0.03 | < 0.01 | . | . |
| OTU 50 Vaginal->OTU 2 Vaginal | < 0.01 | < 0.01 | 0.02 | < 0.01 |
| OTU 50 Vaginal->OTU 3 Vaginal | < 0.01 | < 0.01 | 0.02 | < 0.01 |
| OTU 50 Vaginal->OTU 6 Vaginal | < 0.01 | < 0.01 | < 0.01 | < 0.01 |
| OTU 50 Vaginal->OTU 7 Vaginal | < 0.01 | < 0.01 | 0.01 | < 0.01 |
| OTU 50 Vaginal->OTU 9 Vaginal | < 0.01 | < 0.01 | 0.02 | < 0.01 |
| OTU 52 Fecal->OTU 64 Vaginal | 0.08 | 0.01 | 0.15 | < 0.01 |
| OTU 53 Fecal->OTU 11 Fecal | 0.02 | 0.01 | 0.06 | < 0.01 |
| OTU 53 Fecal->OTU 13 Fecal | 0.06 | < 0.01 | . | < 0.01 |
| OTU 53 Fecal->OTU 29 Fecal | 0.02 | 0.01 | 0.06 | < 0.01 |
| OTU 53 Fecal->OTU 31 Fecal | 0.02 | < 0.01 | 0.04 | < 0.01 |
| OTU 53 Fecal->OTU 42 Fecal | 0.07 | 0.04 | 0.13 | < 0.01 |
| OTU 53 Fecal->OTU 54 Fecal | 0.07 | 0.01 | 0.10 | < 0.01 |
| OTU 53 Fecal->OTU 56 Fecal | 0.04 | 0.01 | . | < 0.01 |
| OTU 53 Fecal->OTU 77 Fecal | 0.10 | < 0.01 | . | < 0.01 |
| OTU 53 Vaginal->OTU 20 Vaginal | 0.02 | < 0.01 | 0.04 | < 0.01 |
| OTU 53 Vaginal->OTU 38 Vaginal | 0.01 | < 0.01 | 0.04 | < 0.01 |
| OTU 53 Vaginal->OTU 68 Vaginal | 0.06 | 0.01 | 0.10 | < 0.01 |
| OTU 54 Fecal->OTU 31 Fecal | 0.01 | < 0.01 | 0.07 | < 0.01 |
| OTU 55 Fecal->OTU 66 Vaginal | 0.02 | 0.01 | 0.08 | < 0.01 |
| OTU 55 Vaginal->OTU 16 Vaginal | 0.01 | < 0.01 | 0.05 | < 0.01 |
| OTU 55 Vaginal->OTU 83 Vaginal | 0.03 | 0.01 | 0.10 | < 0.01 |
| OTU 57 Fecal->OTU 40 Fecal | 0.09 | 0.02 | 0.21 | < 0.01 |
| OTU 57 Fecal->OTU 43 Fecal | < 0.01 | < 0.01 | 0.02 | < 0.01 |
| OTU 57 Fecal->OTU 64 Fecal | < 0.01 | < 0.01 | 0.02 | < 0.01 |
| OTU 57 Fecal->OTU 68 Fecal | < 0.01 | < 0.01 | 0.01 | < 0.01 |
| OTU 57 Fecal->OTU 76 Fecal | < 0.01 | < 0.01 | 0.05 | < 0.01 |
| OTU 57 Fecal->OTU 81 Fecal | < 0.01 | < 0.01 | 0.01 | < 0.01 |
| OTU 57 Fecal->OTU 85 Fecal | < 0.01 | < 0.01 | 0.06 | < 0.01 |
| OTU 57 Fecal->OTU 89 Fecal | < 0.01 | < 0.01 | 0.02 | < 0.01 |
| OTU 57 Fecal->OTU 93 Fecal | 0.06 | 0.03 | 0.17 | < 0.01 |
| OTU 58 Fecal->OTU 33 Fecal | < 0.01 | < 0.01 | 0.03 | < 0.01 |
| OTU 58 Fecal->OTU 36 Vaginal | 0.07 | 0.02 | 0.15 | < 0.01 |
| OTU 60 Vaginal->OTU 30 Vaginal | 0.04 | 0.02 | 0.12 | < 0.01 |
| OTU 61 Fecal->OTU 52 Fecal | 0.12 | < 0.01 | 0.13 | < 0.01 |
| OTU 62 Fecal->OTU 35 Vaginal | 0.07 | 0.10 | 0.14 | < 0.01 |
| OTU 62 Fecal->OTU 52 Fecal | 0.11 | 0.01 | 0.12 | < 0.01 |
| OTU 62 Vaginal->OTU 2 Vaginal | < 0.01 | < 0.01 | 0.06 | < 0.01 |
| OTU 62 Vaginal->OTU 3 Vaginal | < 0.01 | < 0.01 | 0.02 | < 0.01 |
| OTU 62 Vaginal->OTU 6 Vaginal | < 0.01 | < 0.01 | 0.03 | < 0.01 |
| OTU 62 Vaginal->OTU 7 Vaginal | < 0.01 | < 0.01 | 0.02 | < 0.01 |
| OTU 62 Vaginal->OTU 9 Vaginal | < 0.01 | < 0.01 | 0.01 | < 0.01 |
| OTU 62 Vaginal->OTU 14 Vaginal | < 0.01 | < 0.01 | 0.02 | < 0.01 |
| OTU 62 Vaginal->OTU 18 Vaginal | 0.08 | < 0.01 | 0.08 | < 0.01 |
| OTU 62 Vaginal->OTU 22 Vaginal | 0.04 | < 0.01 | 0.15 | < 0.01 |
| OTU 62 Vaginal->OTU 50 Vaginal | < 0.01 | < 0.01 | 0.03 | < 0.01 |
| OTU 62 Vaginal->OTU 78 Vaginal | 0.10 | 0.02 | 0.22 | 0.01 |
| OTU 64 Fecal->OTU 40 Fecal | 0.08 | 0.02 | 0.19 | < 0.01 |
| OTU 64 Fecal->OTU 43 Fecal | < 0.01 | < 0.01 | 0.01 | < 0.01 |
| OTU 64 Fecal->OTU 68 Fecal | < 0.01 | < 0.01 | 0.02 | < 0.01 |
| OTU 64 Fecal->OTU 76 Fecal | < 0.01 | < 0.01 | 0.02 | < 0.01 |
| OTU 64 Fecal->OTU 81 Fecal | < 0.01 | < 0.01 | 0.01 | < 0.01 |
| OTU 64 Fecal->OTU 85 Fecal | < 0.01 | < 0.01 | 0.01 | < 0.01 |
| OTU 64 Fecal->OTU 89 Fecal | < 0.01 | < 0.01 | 0.01 | < 0.01 |
| OTU 64 Fecal->OTU 93 Fecal | 0.08 | 0.05 | . | < 0.01 |
| OTU 64 Vaginal->OTU 30 Vaginal | 0.07 | 0.02 | 0.17 | < 0.01 |
| OTU 64 Vaginal->OTU 88 Vaginal | 0.04 | 0.01 | 0.12 | < 0.01 |
| OTU 64 Vaginal->OTU 97 Vaginal | 0.06 | 0.03 | 0.14 | < 0.01 |
| OTU 65 Fecal->OTU 29 Fecal | 0.06 | 0.01 | 0.11 | < 0.01 |
| OTU 65 Fecal->OTU 31 Fecal | 0.01 | 0.01 | 0.11 | < 0.01 |
| OTU 65 Fecal->OTU 53 Fecal | 0.03 | 0.05 | 0.12 | < 0.01 |
| OTU 65 Fecal->OTU 56 Fecal | 0.03 | 0.01 | 0.09 | < 0.01 |
| OTU 65 Fecal->OTU 77 Fecal | 0.02 | 0.01 | 0.08 | < 0.01 |
| OTU 66 Vaginal->OTU 35 Vaginal | 0.15 | 0.04 | 0.24 | < 0.01 |
| OTU 67 Fecal->OTU 100 Fecal | 0.06 | 0.01 | 0.16 | < 0.01 |
| OTU 67 Fecal->OTU 10 Fecal | 0.03 | 0.01 | 0.19 | < 0.01 |
| OTU 67 Fecal->OTU 25 Fecal | < 0.01 | < 0.01 | < 0.01 | < 0.01 |
| OTU 67 Fecal->OTU 43 Fecal | < 0.01 | < 0.01 | 0.04 | < 0.01 |
| OTU 67 Fecal->OTU 57 Fecal | < 0.01 | < 0.01 | 0.02 | < 0.01 |
| OTU 67 Fecal->OTU 64 Fecal | < 0.01 | < 0.01 | 0.02 | < 0.01 |
| OTU 67 Fecal->OTU 68 Fecal | < 0.01 | < 0.01 | 0.03 | < 0.01 |
| OTU 67 Fecal->OTU 70 Fecal | 0.08 | < 0.01 | 0.09 | < 0.01 |
| OTU 67 Fecal->OTU 74 Fecal | 0.04 | < 0.01 | 0.11 | < 0.01 |
| OTU 67 Fecal->OTU 76 Fecal | 0.01 | < 0.01 | 0.08 | < 0.01 |
| OTU 67 Fecal->OTU 78 Fecal | 0.01 | < 0.01 | 0.08 | < 0.01 |
| OTU 67 Fecal->OTU 81 Fecal | < 0.01 | < 0.01 | 0.01 | < 0.01 |
| OTU 67 Fecal->OTU 85 Fecal | 0.01 | < 0.01 | 0.01 | < 0.01 |
| OTU 67 Fecal->OTU 86 Fecal | < 0.01 | < 0.01 | 0.01 | < 0.01 |
| OTU 67 Fecal->OTU 87 Fecal | 0.01 | < 0.01 | 0.04 | < 0.01 |
| OTU 67 Fecal->OTU 89 Fecal | < 0.01 | < 0.01 | 0.02 | < 0.01 |
| OTU 68 Fecal->OTU 40 Fecal | 0.11 | 0.03 | 0.23 | 0.01 |
| OTU 68 Fecal->OTU 76 Fecal | < 0.01 | < 0.01 | 0.05 | < 0.01 |
| OTU 68 Fecal->OTU 81 Fecal | 0.02 | < 0.01 | 0.05 | < 0.01 |
| OTU 68 Fecal->OTU 85 Fecal | < 0.01 | < 0.01 | 0.04 | < 0.01 |
| OTU 68 Fecal->OTU 93 Fecal | 0.03 | 0.03 | 0.16 | < 0.01 |
| OTU 68 Vaginal->OTU 30 Vaginal | 0.02 | < 0.01 | 0.08 | < 0.01 |
| OTU 68 Vaginal->OTU 38 Vaginal | 0.02 | < 0.01 | 0.10 | < 0.01 |
| OTU 69 Vaginal->OTU 43 Vaginal | < 0.01 | 0.01 | 0.02 | < 0.01 |
| OTU 70 Fecal->OTU 43 Fecal | 0.09 | < 0.01 | . | . |
| OTU 70 Fecal->OTU 57 Fecal | 0.11 | < 0.01 | . | . |
| OTU 70 Fecal->OTU 64 Fecal | 0.03 | < 0.01 | 0.08 | < 0.01 |
| OTU 70 Fecal->OTU 68 Fecal | 0.11 | < 0.01 | . | . |
| OTU 70 Fecal->OTU 81 Fecal | 0.04 | < 0.01 | 0.09 | < 0.01 |
| OTU 70 Fecal->OTU 85 Fecal | 0.04 | < 0.01 | 0.06 | < 0.01 |
| OTU 70 Fecal->OTU 87 Fecal | 0.05 | < 0.01 | 0.08 | < 0.01 |
| OTU 70 Fecal->OTU 89 Fecal | 0.02 | < 0.01 | 0.09 | < 0.01 |
| OTU 71 Fecal->OTU 44 Fecal | 0.06 | < 0.01 | 0.07 | < 0.01 |
| OTU 71 Fecal->OTU 51 Fecal | 0.06 | < 0.01 | 0.06 | < 0.01 |
| OTU 71 Vaginal->OTU 33 Fecal | 0.03 | 0.04 | 0.12 | < 0.01 |
| OTU 71 Vaginal->OTU 36 Vaginal | 0.01 | < 0.01 | 0.05 | < 0.01 |
| OTU 71 Vaginal->OTU 58 Fecal | 0.01 | < 0.01 | 0.04 | < 0.01 |
| OTU 71 Vaginal->OTU 66 Vaginal | 0.10 | 0.02 | 0.20 | < 0.01 |
| OTU 73 Fecal->OTU 44 Fecal | 0.02 | 0.01 | 0.07 | < 0.01 |
| OTU 73 Vaginal->OTU 65 Vaginal | 0.01 | < 0.01 | 0.03 | < 0.01 |
| OTU 74 Fecal->OTU 39 Fecal | 0.06 | 0.01 | 0.14 | < 0.01 |
| OTU 74 Fecal->OTU 40 Fecal | 0.06 | < 0.01 | 0.15 | < 0.01 |
| OTU 74 Fecal->OTU 43 Fecal | 0.02 | < 0.01 | 0.10 | < 0.01 |
| OTU 74 Fecal->OTU 57 Fecal | 0.02 | < 0.01 | 0.11 | < 0.01 |
| OTU 74 Fecal->OTU 64 Fecal | 0.07 | 0.01 | 0.15 | < 0.01 |
| OTU 74 Fecal->OTU 68 Fecal | 0.03 | < 0.01 | 0.10 | < 0.01 |
| OTU 74 Fecal->OTU 76 Fecal | 0.04 | < 0.01 | 0.10 | < 0.01 |
| OTU 74 Fecal->OTU 78 Fecal | < 0.01 | < 0.01 | 0.02 | < 0.01 |
| OTU 74 Fecal->OTU 87 Fecal | . | 0.01 | . | < 0.01 |
| OTU 74 Fecal->OTU 89 Fecal | 0.11 | 0.02 | 0.22 | < 0.01 |
| OTU 74 Fecal->OTU 96 Fecal | 0.07 | 0.03 | 0.14 | < 0.01 |
| OTU 74 Vaginal->OTU 34 Vaginal | 0.05 | 0.01 | 0.07 | < 0.01 |
| OTU 76 Fecal->OTU 93 Fecal | 0.04 | 0.02 | 0.15 | < 0.01 |
| OTU 77 Fecal->OTU 31 Fecal | 0.03 | < 0.01 | 0.10 | < 0.01 |
| OTU 77 Fecal->OTU 56 Fecal | 0.07 | 0.02 | 0.12 | < 0.01 |
| OTU 78 Fecal->OTU 10 Fecal | 0.02 | 0.01 | 0.12 | < 0.01 |
| OTU 78 Fecal->OTU 39 Fecal | < 0.01 | < 0.01 | 0.06 | < 0.01 |
| OTU 78 Fecal->OTU 40 Fecal | 0.01 | < 0.01 | 0.07 | < 0.01 |
| OTU 78 Fecal->OTU 43 Fecal | < 0.01 | < 0.01 | 0.04 | < 0.01 |
| OTU 78 Fecal->OTU 57 Fecal | 0.01 | < 0.01 | 0.07 | < 0.01 |
| OTU 78 Fecal->OTU 64 Fecal | 0.02 | < 0.01 | 0.10 | < 0.01 |
| OTU 78 Fecal->OTU 68 Fecal | < 0.01 | < 0.01 | 0.04 | < 0.01 |
| OTU 78 Fecal->OTU 76 Fecal | < 0.01 | < 0.01 | 0.04 | < 0.01 |
| OTU 78 Fecal->OTU 81 Fecal | 0.04 | 0.02 | 0.15 | < 0.01 |
| OTU 78 Fecal->OTU 85 Fecal | 0.03 | < 0.01 | 0.09 | < 0.01 |
| OTU 78 Fecal->OTU 89 Fecal | 0.04 | 0.01 | 0.15 | < 0.01 |
| OTU 78 Fecal->OTU 93 Fecal | 0.01 | < 0.01 | 0.08 | < 0.01 |
| OTU 78 Vaginal->OTU 2 Vaginal | 0.06 | < 0.01 | 0.21 | 0.01 |
| OTU 78 Vaginal->OTU 3 Vaginal | 0.03 | < 0.01 | 0.20 | < 0.01 |
| OTU 78 Vaginal->OTU 6 Vaginal | 0.03 | < 0.01 | 0.19 | < 0.01 |
| OTU 78 Vaginal->OTU 7 Vaginal | 0.05 | 0.01 | 0.22 | 0.01 |
| OTU 78 Vaginal->OTU 9 Vaginal | 0.04 | 0.01 | 0.21 | < 0.01 |
| OTU 78 Vaginal->OTU 14 Vaginal | 0.04 | 0.01 | 0.21 | < 0.01 |
| OTU 78 Vaginal->OTU 18 Vaginal | 0.06 | 0.02 | 0.21 | < 0.01 |
| OTU 78 Vaginal->OTU 22 Vaginal | 0.01 | < 0.01 | 0.06 | < 0.01 |
| OTU 78 Vaginal->OTU 43 Vaginal | 0.01 | < 0.01 | 0.05 | < 0.01 |
| OTU 78 Vaginal->OTU 47 Vaginal | 0.09 | < 0.01 | . | . |
| OTU 78 Vaginal->OTU 65 Vaginal | 0.04 | 0.01 | 0.09 | < 0.01 |
| OTU 78 Vaginal->OTU 73 Vaginal | 0.05 | < 0.01 | 0.11 | < 0.01 |
| OTU 78 Vaginal->OTU 81 Fecal | 0.13 | 0.03 | . | < 0.01 |
| OTU 80 Vaginal->OTU 26 Vaginal | 0.02 | < 0.01 | 0.05 | < 0.01 |
| OTU 80 Vaginal->OTU 27 Vaginal | 0.08 | . | 0.16 | < 0.01 |
| OTU 80 Vaginal->OTU 30 Vaginal | 0.02 | < 0.01 | 0.05 | < 0.01 |
| OTU 80 Vaginal->OTU 60 Vaginal | < 0.01 | < 0.01 | 0.02 | < 0.01 |
| OTU 80 Vaginal->OTU 83 Vaginal | 0.03 | 0.02 | 0.09 | < 0.01 |
| OTU 80 Vaginal->OTU 97 Vaginal | 0.05 | 0.01 | 0.09 | < 0.01 |
| OTU 81 Fecal->OTU 76 Fecal | 0.01 | < 0.01 | 0.07 | < 0.01 |
| OTU 81 Fecal->OTU 85 Fecal | < 0.01 | < 0.01 | 0.01 | < 0.01 |
| OTU 82 Fecal->OTU 27 Fecal | . | < 0.01 | . | . |
| OTU 82 Fecal->OTU 52 Fecal | 0.01 | < 0.01 | 0.03 | < 0.01 |
| OTU 82 Fecal->OTU 61 Fecal | 0.05 | < 0.01 | 0.07 | < 0.01 |
| OTU 82 Fecal->OTU 64 Vaginal | 0.10 | 0.03 | . | < 0.01 |
| OTU 83 Fecal->OTU 7 Fecal | 0.11 | 0.01 | 0.17 | < 0.01 |
| OTU 83 Fecal->OTU 11 Fecal | 0.10 | 0.01 | . | . |
| OTU 83 Fecal->OTU 13 Fecal | 0.15 | 0.01 | . | . |
| OTU 83 Fecal->OTU 29 Fecal | 0.08 | < 0.01 | . | . |
| OTU 83 Fecal->OTU 31 Fecal | < 0.01 | < 0.01 | 0.04 | < 0.01 |
| OTU 83 Fecal->OTU 54 Fecal | 0.04 | 0.01 | 0.06 | < 0.01 |
| OTU 83 Fecal->OTU 56 Fecal | 0.04 | 0.01 | 0.12 | < 0.01 |
| OTU 83 Fecal->OTU 65 Fecal | 0.05 | 0.01 | 0.13 | < 0.01 |
| OTU 83 Fecal->OTU 77 Fecal | 0.01 | < 0.01 | 0.04 | < 0.01 |
| OTU 83 Vaginal->OTU 30 Vaginal | 0.04 | 0.01 | 0.13 | < 0.01 |
| OTU 83 Vaginal->OTU 60 Vaginal | 0.10 | 0.01 | 0.15 | < 0.01 |
| OTU 83 Vaginal->OTU 92 Vaginal | 0.03 | < 0.01 | 0.05 | < 0.01 |
| OTU 83 Vaginal->OTU 93 Vaginal | 0.01 | 0.01 | 0.05 | < 0.01 |
| OTU 85 Fecal->OTU 76 Fecal | 0.03 | 0.01 | 0.10 | < 0.01 |
| OTU 86 Fecal->OTU 100 Fecal | 0.04 | 0.01 | 0.11 | < 0.01 |
| OTU 86 Fecal->OTU 10 Fecal | 0.11 | < 0.01 | 0.21 | 0.01 |
| OTU 86 Fecal->OTU 25 Fecal | < 0.01 | < 0.01 | 0.01 | < 0.01 |
| OTU 86 Fecal->OTU 43 Fecal | 0.01 | < 0.01 | 0.06 | < 0.01 |
| OTU 86 Fecal->OTU 57 Fecal | < 0.01 | < 0.01 | 0.02 | < 0.01 |
| OTU 86 Fecal->OTU 64 Fecal | < 0.01 | < 0.01 | 0.02 | < 0.01 |
| OTU 86 Fecal->OTU 68 Fecal | 0.01 | < 0.01 | 0.06 | < 0.01 |
| OTU 86 Fecal->OTU 70 Fecal | 0.03 | < 0.01 | 0.09 | < 0.01 |
| OTU 86 Fecal->OTU 74 Fecal | 0.07 | < 0.01 | 0.13 | < 0.01 |
| OTU 86 Fecal->OTU 76 Fecal | 0.01 | < 0.01 | 0.09 | < 0.01 |
| OTU 86 Fecal->OTU 78 Fecal | 0.07 | < 0.01 | 0.14 | < 0.01 |
| OTU 86 Fecal->OTU 81 Fecal | < 0.01 | < 0.01 | 0.02 | < 0.01 |
| OTU 86 Fecal->OTU 85 Fecal | < 0.01 | < 0.01 | 0.02 | < 0.01 |
| OTU 86 Fecal->OTU 87 Fecal | < 0.01 | < 0.01 | 0.05 | < 0.01 |
| OTU 86 Fecal->OTU 89 Fecal | < 0.01 | < 0.01 | 0.02 | < 0.01 |
| OTU 87 Fecal->OTU 40 Fecal | 0.19 | 0.03 | . | < 0.01 |
| OTU 87 Fecal->OTU 43 Fecal | 0.06 | < 0.01 | 0.17 | < 0.01 |
| OTU 87 Fecal->OTU 57 Fecal | 0.01 | < 0.01 | 0.10 | < 0.01 |
| OTU 87 Fecal->OTU 64 Fecal | 0.01 | < 0.01 | 0.08 | < 0.01 |
| OTU 87 Fecal->OTU 68 Fecal | 0.04 | 0.01 | 0.16 | < 0.01 |
| OTU 87 Fecal->OTU 76 Fecal | 0.06 | 0.01 | 0.14 | < 0.01 |
| OTU 87 Fecal->OTU 78 Fecal | 0.10 | 0.01 | 0.19 | < 0.01 |
| OTU 87 Fecal->OTU 81 Fecal | 0.01 | < 0.01 | 0.09 | < 0.01 |
| OTU 87 Fecal->OTU 85 Fecal | 0.03 | < 0.01 | 0.04 | < 0.01 |
| OTU 87 Fecal->OTU 89 Fecal | < 0.01 | < 0.01 | 0.05 | < 0.01 |
| OTU 87 Vaginal->OTU 68 Vaginal | . | . | . | < 0.01 |
| OTU 87 Vaginal->OTU 99 Vaginal | . | . | . | < 0.01 |
| OTU 88 Fecal->OTU 61 Fecal | 0.02 | < 0.01 | 0.05 | < 0.01 |
| OTU 88 Vaginal->OTU 30 Vaginal | 0.08 | 0.01 | . | < 0.01 |
| OTU 88 Vaginal->OTU 52 Fecal | 0.10 | 0.02 | 0.13 | < 0.01 |
| OTU 88 Vaginal->OTU 67 Vaginal | 0.09 | 0.02 | 0.12 | < 0.01 |
| OTU 88 Vaginal->OTU 97 Vaginal | < 0.01 | < 0.01 | 0.01 | < 0.01 |
| OTU 89 Fecal->OTU 43 Fecal | < 0.01 | < 0.01 | 0.03 | < 0.01 |
| OTU 89 Fecal->OTU 68 Fecal | < 0.01 | < 0.01 | 0.03 | < 0.01 |
| OTU 89 Fecal->OTU 76 Fecal | < 0.01 | < 0.01 | 0.04 | < 0.01 |
| OTU 89 Fecal->OTU 81 Fecal | < 0.01 | < 0.01 | 0.01 | < 0.01 |
| OTU 89 Fecal->OTU 85 Fecal | < 0.01 | < 0.01 | 0.01 | < 0.01 |
| OTU 89 Fecal->OTU 93 Fecal | 0.10 | 0.06 | . | < 0.01 |
| OTU 90 Fecal->OTU 94 Vaginal | < 0.01 | < 0.01 | < 0.01 | < 0.01 |
| OTU 91 Fecal->OTU 7 Fecal | 0.07 | 0.03 | 0.15 | < 0.01 |
| OTU 92 Fecal->OTU 100 Fecal | 0.05 | 0.01 | 0.16 | < 0.01 |
| OTU 92 Fecal->OTU 10 Fecal | 0.05 | < 0.01 | 0.16 | < 0.01 |
| OTU 92 Fecal->OTU 20 Fecal | 0.03 | < 0.01 | 0.11 | < 0.01 |
| OTU 92 Fecal->OTU 25 Fecal | < 0.01 | < 0.01 | 0.09 | < 0.01 |
| OTU 92 Fecal->OTU 39 Fecal | 0.01 | 0.01 | 0.11 | < 0.01 |
| OTU 92 Fecal->OTU 40 Fecal | < 0.01 | < 0.01 | 0.01 | < 0.01 |
| OTU 92 Fecal->OTU 43 Fecal | 0.03 | < 0.01 | 0.14 | < 0.01 |
| OTU 92 Fecal->OTU 57 Fecal | 0.01 | < 0.01 | 0.06 | < 0.01 |
| OTU 92 Fecal->OTU 64 Fecal | 0.02 | < 0.01 | 0.09 | < 0.01 |
| OTU 92 Fecal->OTU 67 Fecal | 0.06 | 0.01 | 0.16 | < 0.01 |
| OTU 92 Fecal->OTU 68 Fecal | 0.02 | < 0.01 | 0.12 | < 0.01 |
| OTU 92 Fecal->OTU 74 Fecal | 0.03 | < 0.01 | 0.08 | < 0.01 |
| OTU 92 Fecal->OTU 76 Fecal | < 0.01 | < 0.01 | 0.02 | < 0.01 |
| OTU 92 Fecal->OTU 78 Fecal | < 0.01 | < 0.01 | 0.03 | < 0.01 |
| OTU 92 Fecal->OTU 81 Fecal | 0.08 | 0.02 | 0.20 | < 0.01 |
| OTU 92 Fecal->OTU 85 Fecal | 0.09 | 0.02 | 0.18 | < 0.01 |
| OTU 92 Fecal->OTU 86 Fecal | 0.09 | < 0.01 | 0.15 | < 0.01 |
| OTU 92 Fecal->OTU 87 Fecal | 0.11 | 0.01 | 0.16 | < 0.01 |
| OTU 92 Fecal->OTU 89 Fecal | 0.05 | 0.01 | 0.13 | < 0.01 |
| OTU 92 Fecal->OTU 93 Fecal | 0.09 | 0.01 | 0.14 | < 0.01 |
| OTU 92 Fecal->OTU 96 Fecal | 0.01 | < 0.01 | 0.07 | < 0.01 |
| OTU 93 Vaginal->OTU 92 Vaginal | 0.01 | 0.05 | 0.06 | < 0.01 |
| OTU 96 Fecal->OTU 39 Fecal | 0.05 | 0.06 | 0.18 | < 0.01 |
| OTU 96 Fecal->OTU 40 Fecal | < 0.01 | < 0.01 | 0.03 | < 0.01 |
| OTU 96 Fecal->OTU 57 Fecal | 0.12 | 0.02 | 0.19 | < 0.01 |
| OTU 96 Fecal->OTU 64 Fecal | 0.09 | 0.05 | 0.20 | < 0.01 |
| OTU 96 Fecal->OTU 76 Fecal | 0.02 | 0.01 | 0.11 | < 0.01 |
| OTU 96 Fecal->OTU 78 Fecal | 0.03 | 0.01 | 0.10 | < 0.01 |
| OTU 96 Fecal->OTU 93 Fecal | 0.12 | 0.02 | 0.16 | < 0.01 |
| OTU 97 Vaginal->OTU 30 Vaginal | 0.03 | < 0.01 | 0.08 | < 0.01 |
| OTU 97 Vaginal->OTU 60 Vaginal | 0.10 | 0.01 | 0.14 | < 0.01 |
| OTU 98 Fecal->OTU 21 Fecal | 0.03 | 0.06 | . | < 0.01 |
| OTU 98 Fecal->OTU 44 Fecal | 0.01 | 0.01 | . | < 0.01 |
| OTU 98 Fecal->OTU 46 Fecal | 0.23 | . | . | < 0.01 |
| OTU 98 Fecal->OTU 69 Fecal | 0.11 | 0.05 | . | < 0.01 |
| OTU 98 Fecal->OTU 73 Fecal | 0.01 | < 0.01 | . | < 0.01 |
| OTU 100 Fecal->OTU 43 Fecal | < 0.01 | < 0.01 | 0.07 | < 0.01 |
| OTU 100 Fecal->OTU 57 Fecal | < 0.01 | < 0.01 | 0.04 | < 0.01 |
| OTU 100 Fecal->OTU 64 Fecal | 0.01 | < 0.01 | 0.08 | < 0.01 |
| OTU 100 Fecal->OTU 68 Fecal | < 0.01 | < 0.01 | 0.04 | < 0.01 |
| OTU 100 Fecal->OTU 76 Fecal | 0.01 | 0.01 | 0.10 | < 0.01 |
| OTU 100 Fecal->OTU 78 Fecal | 0.05 | 0.02 | 0.18 | < 0.01 |
| OTU 100 Fecal->OTU 81 Fecal | 0.03 | 0.01 | 0.11 | < 0.01 |
| OTU 100 Fecal->OTU 85 Fecal | 0.07 | 0.01 | 0.19 | < 0.01 |
| OTU 100 Fecal->OTU 87 Fecal | 0.09 | 0.03 | 0.23 | < 0.01 |
| OTU 100 Fecal->OTU 89 Fecal | 0.02 | < 0.01 | 0.13 | < 0.01 |
| ^1^ Nodes represent the Operational Taxonomic Units (OTU’s) of either the fecal or vaginal communities of late gestation sows | | | | |
| ^2^ Label of the specific node that is significantly correlated to the second  ^3^ Pearson correlation  ^4^ Spearman correlation  ^5^ Bray Curtis dissimilarity  ^6^ Kullback-Leibler Dissimilarity  . denotes missing value or calculation was not considered significant | | | | |
